# Expression Dysregulation as a Mediator of Fitness Costs in Antibiotic Resistance

**DOI:** 10.1128/AAC.00504-21

**Published:** 2021-08-17

**Authors:** Andrej Trauner, Amir Banaei-Esfahani, Sebastian M. Gygli, Philipp Warmer, Julia Feldmann, Mattia Zampieri, Sonia Borrell, Ben C. Collins, Christian Beisel, Ruedi Aebersold, Sebastien Gagneux

**Affiliations:** a Swiss Tropical and Public Health Institute, Basel, Switzerland; b University of Basel, Basel, Switzerland; c Department of Biology, Institute of Molecular and Systems Biology, ETH Zurich, Zurich, Switzerland; d PhD Program in Systems Biology, Life Science Zurich Graduate School, University of Zurich and ETH Zurich, Zurich, Switzerland; e Genomics Facility Basel, Department of Biosystems Science and Engineering, ETH Zurich, Basel, Switzerland; f Faculty of Science, University of Zurich, Zurich, Switzerland

**Keywords:** antibiotic resistance, evolution, fitness, gene expression, mycobacteria, proteomics, transcription, tuberculosis

## Abstract

Antimicrobial resistance (AMR) poses a threat to global health and the economy. Rifampicin-resistant Mycobacterium tuberculosis accounts for a third of the global AMR burden. Gaining the upper hand on AMR requires a deeper understanding of the physiology of resistance. AMR often results in a fitness cost in the absence of drug. Identifying the molecular mechanisms underpinning this cost could help strengthen future treatment regimens. Here, we used a collection of M. tuberculosis strains that provide an evolutionary and phylogenetic snapshot of rifampicin resistance and subjected them to genome-wide transcriptomic and proteomic profiling to identify key perturbations of normal physiology. We found that the clinically most common rifampicin resistance-conferring mutation, RpoB Ser450Leu, imparts considerable gene expression changes, many of which are mitigated by the compensatory mutation in RpoC Leu516Pro. However, our data also provide evidence for pervasive epistasis—the same resistance mutation imposed a different fitness cost and functionally distinct changes to gene expression in genetically unrelated clinical strains. Finally, we report a likely posttranscriptional modulation of gene expression that is shared in most of the tested strains carrying RpoB Ser450Leu, resulting in an increased abundance of proteins involved in central carbon metabolism. These changes contribute to a more general trend in which the disruption of the composition of the proteome correlates with the fitness cost of the RpoB Ser450Leu mutation in different strains.

## INTRODUCTION

Antimicrobials are one of the cornerstones of modern medicine (1). The global increase of antimicrobial resistance (AMR) is claiming an increasing number of lives and resources (2). We currently have access to a wide array of antibiotics, but their efficacy is waning, making safeguarding current and future drugs a high priority. Understanding the mechanisms and drivers of AMR will be key to that process (3).

Antibiotics target essential bacterial functions. Modification of those targets is an important mechanism through which AMR emerges. It is therefore not surprising that AMR is often associated with a decreased bacterial growth rate *in vitro*, and in the case of pathogens, a decreased ability to cause disease or transmit in the clinic. These phenomena are commonly referred to as a “fitness cost of drug resistance” (4). The physiological basis for the cost of drug resistance depends on the antibiotic, the bacterial species, and the environment (5) and is thus often unknown and likely to be multifaceted. One of the better studied examples is the cost of rifampicin resistance (6). Rifampicin targets the bacterial RNA polymerase (RNAP), and resistance to rifampicin is usually mediated by mutations in the β subunit of RNAP encoded by *rpoB* (7). Due to its position at the root of gene expression, mutations in RNAP affect which genes are transcribed and their level of expression. Several studies point to the rate of transcription as an important mediator of bacterial growth rate in culture (8, 9). The relative contribution of lower transcriptional efficiency, as opposed to a pleiotropic disruption of gene expression, was therefore suggested as the most likely explanation for the cost of rifampicin resistance (8–10). The mechanism linking RNAP activity to ribosome biosynthesis, and more broadly to the rate of transcription, provides a compelling explanation for the cost of rifampicin resistance in rapidly dividing bacteria grown in optimal conditions, such as Escherichia coli and Pseudomonas aeruginosa, whose growth relies on the rapid replenishment of biosynthetic machinery lost through cell division (11). However, the pressure to replenish ribosomes and proteins essential for rapid growth may be less severe in slow-growing organisms such as Mycobacterium tuberculosis. Thus, alternatively, indirect effects linked to broader changes in gene expression might be responsible for the fitness cost of rifampicin resistance in this organism (6, 12).

Rifampicin-resistant M. tuberculosis is one of the major causes of AMR-associated mortality globally, claiming an estimated 240,000 lives in 2019 (13). As with E. coli or P. aeruginosa, *rpoB* mutations confer resistance and modify the structural and biochemical properties of M. tuberculosis RNAP (14, 15). Importantly, these biochemical changes can be mitigated through the acquisition of secondary, compensatory mutations in the α, β, and β′ subunits of RNAP (8, 10, 15). However, unlike in fast-growing bacteria, the rate of transcription does not seem to reflect the fitness cost of resistance-conferring *rpoB* mutations (see Fig. S1 in the supplemental material), measured either by growth rate *in vitro* or prevalence in the clinic (15–17). Instead, aberrant production of the sole M. tuberculosis siderophore, mycobactin (18), and modification of virulence-modulating lipid phthiocerol dimycocerosate (PDIM) (19) and structural mycolic acids (20) have all been reported in rifampicin-resistant M. tuberculosis, potentially impacting its virulence (21). While these changes suggest that dysregulation of gene expression might be an important consequence of *rpoB* mutations, it remains unclear whether such changes also impart the fitness cost associated with rifampicin resistance in M. tuberculosis.

**FIG 1:**
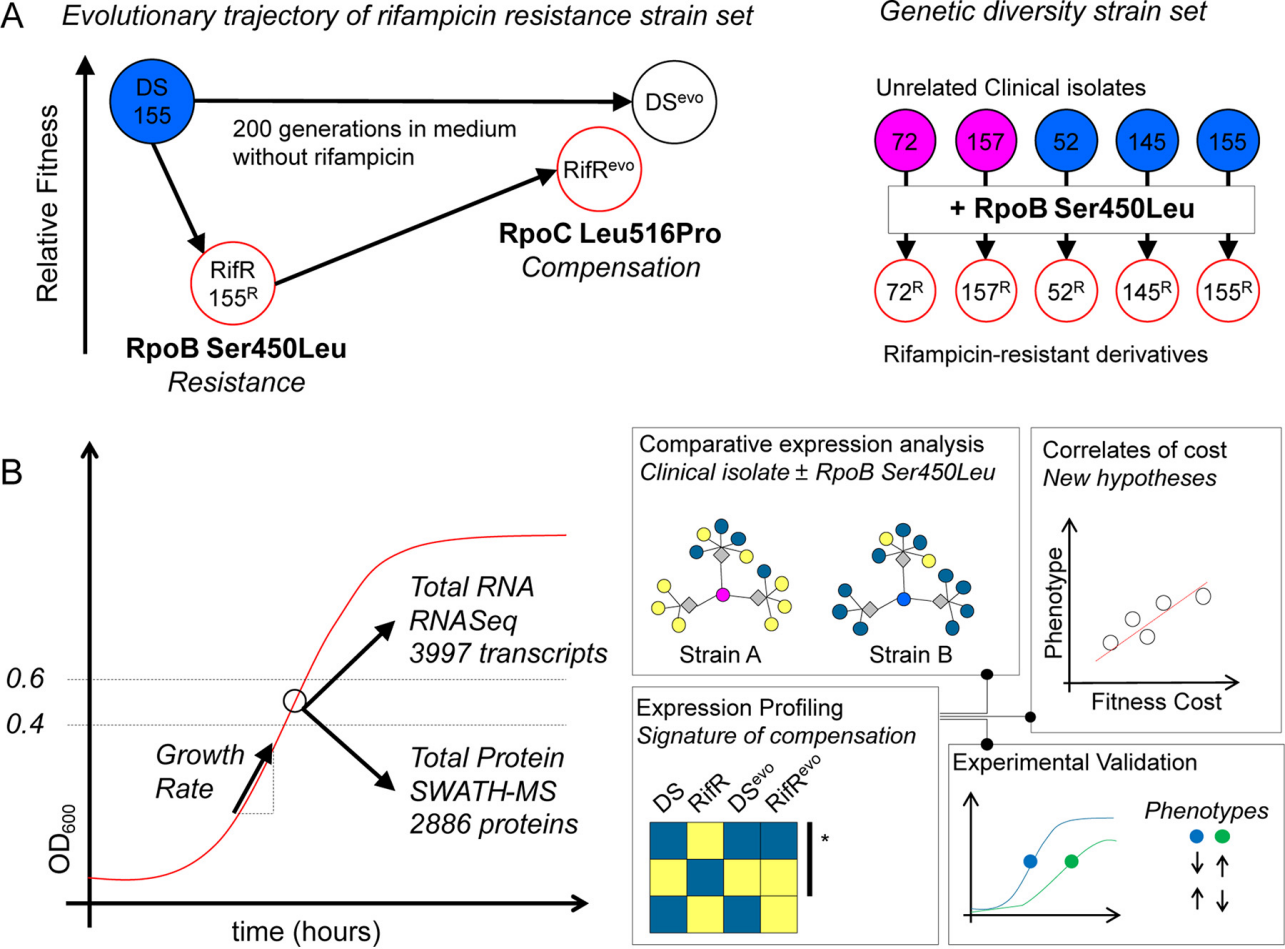
Conceptual workflow. (A) Two complementary strain sets used for the experiments. Strains comprising the “evolutionary trajectory of rifampicin resistance” set were derived from a single clinical isolate (N0155 [DS]) by isolation of a Ser450Leu mutant in the laboratory and subsequent passage for 200 generations in the absence of rifampicin. These strains were used to identify expression changes that are reversed by compensation, the “signature of compensation.” The robustness of our findings was checked using the “genetic diversity strain set,” which contained five independent clinical isolates and their rifampicin-resistant derivatives. All rifampicin-resistant strains shared the same resistance mutation, RpoB Ser450Leu. (B) Experimental outline for sampling and analyses.

We used the known ability of mutations in the β barrel double ψ (BBDP) domain of the β′ subunit of RNAP to compensate for the fitness cost of resistance mutations occurring in the β subunit in M. tuberculosis as a starting point (10, 14, 15). Compensatory mutations improve patient-to-patient transmission of rifampicin-resistant strains (22–24) and partially reverse biochemical changes imparted on RNAP by rifampicin-resistance mutations (10, 15). We hypothesized that the same would be true for gene expression differences (12, 25). Leveraging the knowledge of the role of RpoC mutations, we used transcriptomic and proteomic expression profiling to identify the signature of compensation and therefore to infer the likely mediators of fitness cost in a collection of strains derived from a drug-susceptible clinical isolate (Fig. 1). Our findings point to the idiosyncratic consequences of expressional dysregulation as a key factor in reducing the growth rate of M. tuberculosis in culture, resulting in what we define as the fitness cost of rifampicin resistance in M. tuberculosis. Given that RNAP is largely conserved across M. tuberculosis lineages (26), we posited that the same *rpoB* mutation should have comparable effects in different genetic contexts. We tested the potential generalized mechanism by profiling the expression signature of rifampicin resistance in a panel of genetically diverse clinical strains sharing the same rifampicin resistance-conferring mutation, RpoB Ser450Leu. We found little evidence of a shared transcriptional signature of rifampicin resistance across strains, indicating a strong influence of strain genotypes on the phenotype. In contrast, we observed an association between the fitness cost of the rifampicin resistance-conferring mutation and the extent to which its presence imparted a deviation from the proteome composition of the wild-type strain. While the causality of these changes remains to be established, our findings highlight the importance of gene expression dysregulation as a modulator of normal RNAP and M. tuberculosis physiology.

## RESULTS

### Compensatory mutations mitigate resistance-imposed expression changes.

Physiological changes manifesting as a fitness cost are likely to stem from deviations in gene expression. Since mutations in the BBDP domain of the β′ subunit of RNAP mitigate the fitness cost of rifampicin-resistance mutations in M. tuberculosis (10, 14, 15), they should also impact, and therefore, highlight expression changes that are relevant to the understanding of fitness cost of rifampicin resistance. Indeed, reversal of expression changes in the course of evolutionary adaptation has been shown before (12, 25).

We previously reported the results of a directed evolution experiment in which we identified a mutation in the BBDP domain, RpoC Leu516Pro, as a putative compensatory mechanism for the fitness cost of the rifampicin-resistance conferring mutation RpoB Ser450Leu in a clinical isolate of M. tuberculosis (17, 27). The strains generated by that study comprised the original drug-susceptible isolate (DS), its laboratory-derived rifampicin-resistant mutant (RpoB Ser450Leu) (RifR), and the resulting evolved strains obtained by serial passage in the absence of rifampicin for 200 generations (DSevo and RifRevo), respectively (Fig. 1A). Together, these strains offer a representative snapshot of the evolutionary process prevalent in the clinic that passes through the initial emergence of (costly) drug resistance and leads to the establishment of a mature drug-resistant strain whose fitness is indistinguishable from that of its drug-susceptible ancestor (27). We therefore hypothesized that comparative transcriptomic and proteomic expression profiling of these strains will allow us to determine the expression signature of the fitness cost associated with rifampicin resistance. Based on previous reports, we expected expression changes to fall into two groups. Specifically, we expected to see either an increased expression of RNAP with limited pleiotropic changes, as has been highlighted for E. coli and P. aeruginosa (8, 9), or a shift toward different biosynthetic programs, as reported by studies in M. tuberculosis and Streptomyces (6, 18, 19).

First, we determined the relative fitness of the RifR strain. Using a mixed-effects linear regression model to analyze growth assays, we noted a 26.4% decrease (95% confidence interval [CI], 21.5 to 31.0%; *P* < 0.001) in the growth rate of RifR compared to that of the DS strain. The comparison of their evolved counterparts, DSevo and RifRevo, showed no significant differences (−1.2%; 95% CI, −10.8 to 7.1%; *P* = 0.814), illustrating the fact that RpoC Leu516Pro does indeed compensate the fitness cost of rifampicin resistance.

We aimed to identify differences in the baseline, unperturbed, gene expression as a proxy for describing the biological basis for reduced fitness in RifR. We sampled actively growing bacterial cultures of each of the four strains, extracting total RNA and protein to be profiled using RNA sequencing (RNAseq) and sequential window acquisition of all theoretical mass spectra (SWATH-MS), respectively (Fig. 1B). In total, we were able to obtain RNA transcript counts for all regions of the M. tuberculosis genome and reliably quantify 2,886 proteins across our samples (see Fig. S2 and S3 in the supplemental material). We used differential expression analysis to test our hypothesis that the compensatory mutation RpoC Leu516Pro had the net effect of reversing, at least partially, the expression changes brought about by the rifampicin resistance mutation RpoB Ser450Leu. To address this question, we chose an inclusive definition of differential expression, namely, a *P* value of less than 0.05 after adjusting for multiple testing (see Materials and Methods). In keeping with our inclusive approach, we also deliberately did not use an effect size threshold (e.g., minimum log fold change).

**FIG 2:**
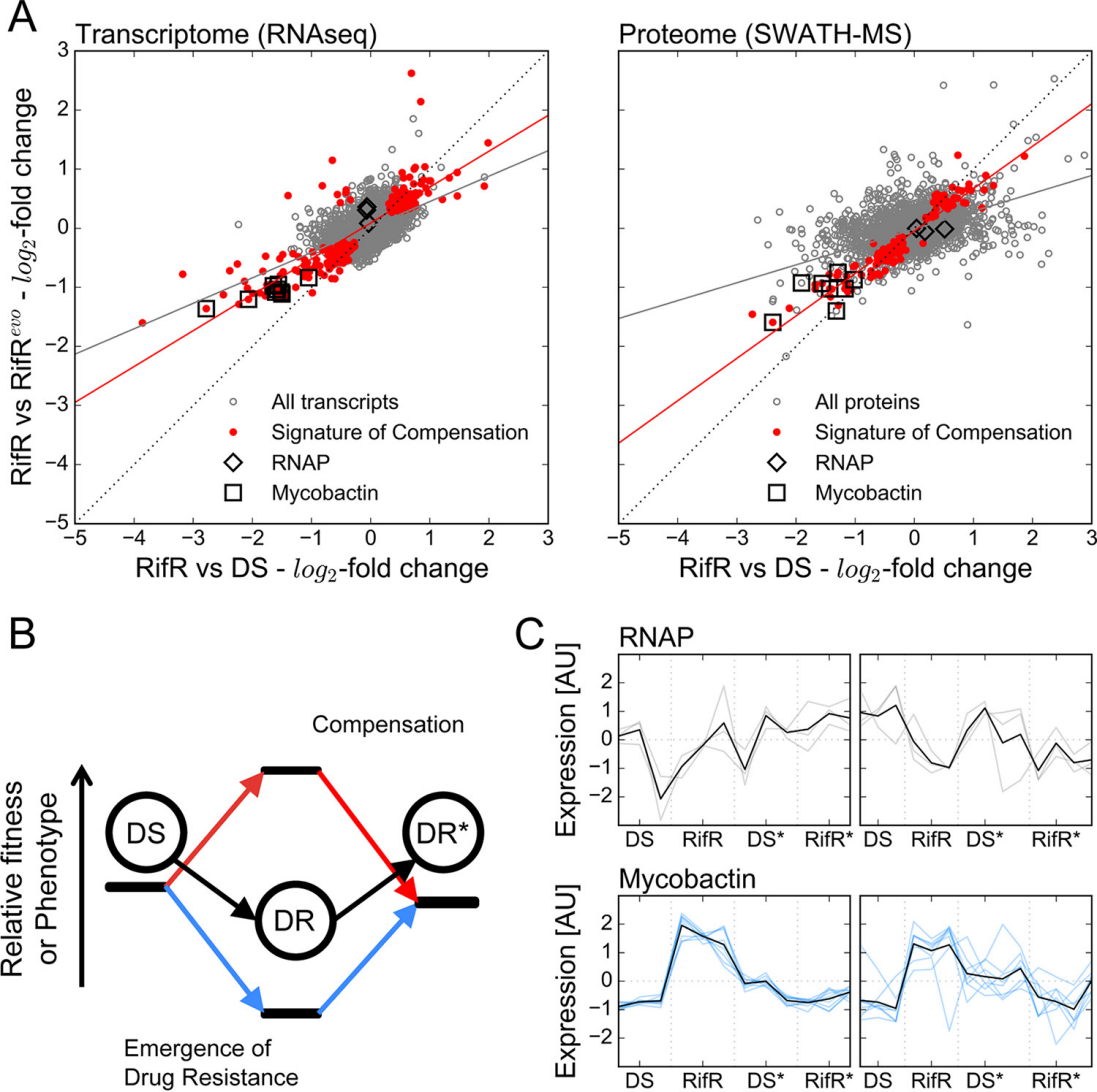
Signature of compensation. (A) Expression changes in RifR and their compensation. Gray dots, all data; red dots, signature of compensation; black diamonds, RNAP genes; black squares, mycobactin genes. The gray line shows a linear regression model fit for all data (slope = 0.43 for RNA and 0.30 for protein; *P* < 0.001 for both). The red line shows a linear regression model fit for “signature of compensation” genes (slope = 0.72 for RNA and 0.60 for protein; *P* < 0.001 for both). (B) The relative fitness of drug-resistant (DR) strains is expected to be lower than that of wild-type (DS) strains at first, but then is expected to increase due to compensatory evolution. The phenotypic equivalent of this trend is illustrated as an increase/decrease in a measurable trait upon the emergence of resistance, which is then returned to its previous level through compensation. We refer to this dynamic as the signature of compensation. (C) Plot of transcript counts per million bases (TPM) and label-free quantifications (LFQ) of cellular proteins for genes whose expression was perturbed by the Ser450Leu mutation in RpoB and returned to the wild type in the presence of the compensating Leu516Pro mutation. All results were standardized across measurements for a single gene to allow comparison between strains. Gray traces show transcripts (left) and proteins (right) of RNAP components (*rpoA*, *rpoB*, and *rpoC*); blue traces show transcripts (left) and proteins (right) of components of the mycobactin biosynthesis cluster. The bold black lines show the mean of the sample.

First, we compared RifR expression to that of its ancestor DS. We found no evidence of changes in the expression of RNAP components (RpoA/B/C) at the level of transcripts or proteins (Fig. 2). Instead, we identified 744/3,976 differentially expressed transcripts, corresponding to 18.7% of the transcriptome, with a median expression change of 47% (interquartile range [IQR] 37 to 63%). Of these, 73 genes showed a dysregulation of 2-fold or more. We observed the disruption of a greater proportion of the proteome in RifR, namely, 998/2,886 differentially expressed proteins (34.6%) showing a similar median expression difference of 45% (IQR, 29 to 78%). Consistently, a greater number of proteins (*N* = 176) exhibited an expression disruption of more than 2-fold. Comparing RifR and RifRevo, we conclude that the RpoC Leu516Pro mutation reversed the dysregulation of 229/744 transcripts (30.8%) and 217/998 proteins (21.7%). Using linear regression to compare the magnitude of gene dysregulation (25), we found that the overall impact of compensation was as expected—to mitigate the changes in normal expression. RpoC Leu516Pro restored, on average, 43% (ordinary least-squares [OLS] linear regression; *P* < 0.001) of the transcriptional and 30% (OLS linear regression, *P* < 0.001) of the knock-on proteomic differences imparted by RpoB Ser450Leu. The smaller magnitude of the latter is reflected in the fact that the proteome of RifRevo is closer to that of RifR than those of DSevo or DS (see Fig. S4 in the supplemental material). Nonetheless, while compensation significantly affected only a minority of the aberrantly expressed genes, it restored 60% (OLS linear regression; *P* < 0.001) of the normal expression in compensated transcripts and 72% (OLS linear regression; *P* < 0.001) of affected proteins (see supplemental material for a more detailed explanation).

### Expression dysregulation as a mediator of fitness cost.

Since RifRevo no longer had a growth defect, despite only partially restoring normal expression, we sought to identify which expression changes were most likely to impart a growth defect on RifR. To this end, we identified genes that were uniquely differentially expressed in RifR compared to the other three strains in our data set and collectively labeled them a “signature of compensation.” Gene set enrichment analysis of the transcriptomic data pointed to iron homeostasis being significantly affected. Specifically, it indicated a higher expression, in RifR, of genes that were repressed by the iron-dependent regulator (IdeR, Rv2711) under iron-replete conditions (Fisher’s exact test; odds ratio = 4.27; *P* = 8.20 × 10^−7^). Among them, there was a significant enrichment of genes involved in polyketide and nonribosomal peptide synthesis (Fisher’s exact test; odds ratio = 3.19; *P* = 2.30 × 10^−4^), which included the biosynthetic machinery for the sole M. tuberculosis siderophore: mycobactin. We found a similar pattern, albeit less pronounced, in the proteomic data (see supplemental note and Fig. S5 in the supplemental material). Together, these changes recapitulate published data (18) and suggest that RifR faced a shortage of iron under our experimental conditions.

The availability of iron is an essential requirement for M. tuberculosis growth, both in culture and during infection, and iron acquisition systems are therefore key virulence factors (28–30). Hence, an increased requirement for iron could manifest itself as a loss of relative fitness. The fact that RpoB Ser450Leu led to a modification of the expression of genes involved in iron homeostasis, and that RpoC Leu516Pro reversed the effect, provides a compelling alternative mechanism underpinning the fitness cost of rifampicin resistance. If the disruption of iron homeostasis drives fitness cost (decreases bacterial growth rates), we would expect that iron supplementation should mitigate the relative cost of RpoB Ser450Leu. Furthermore, based on the expression profile, we expected that RifR should produce more mycobactin at baseline than DS, potentially influencing the overall growth rate of the mutant.

We addressed the first hypothesis by comparing the growth rates of RifR and DS in the presence or absence of 10 μM hemin, an additional source of iron that is by itself sufficient to support the growth of a mutant defective in mycobactin biosynthesis (28). Importantly, hemin and mycobactin provide two separate routes of iron uptake, which allows us to sidestep issues that might emerge from deficient iron transport (28). The presence of hemin did not change the cost of RifR, which we calculated to be 18.6% in the absence and 20.9% in the presence of hemin for this experiment (mixed effects linear model; *P* = 0.737). Similarly, hemin did not impact the growth rate of DS (−4.7%; 95%CI, −16.3 to 2.3%; *P* = 0.128). In summary, iron did not appear to limit the growth of RifR under our conditions.

**FIG 3:**
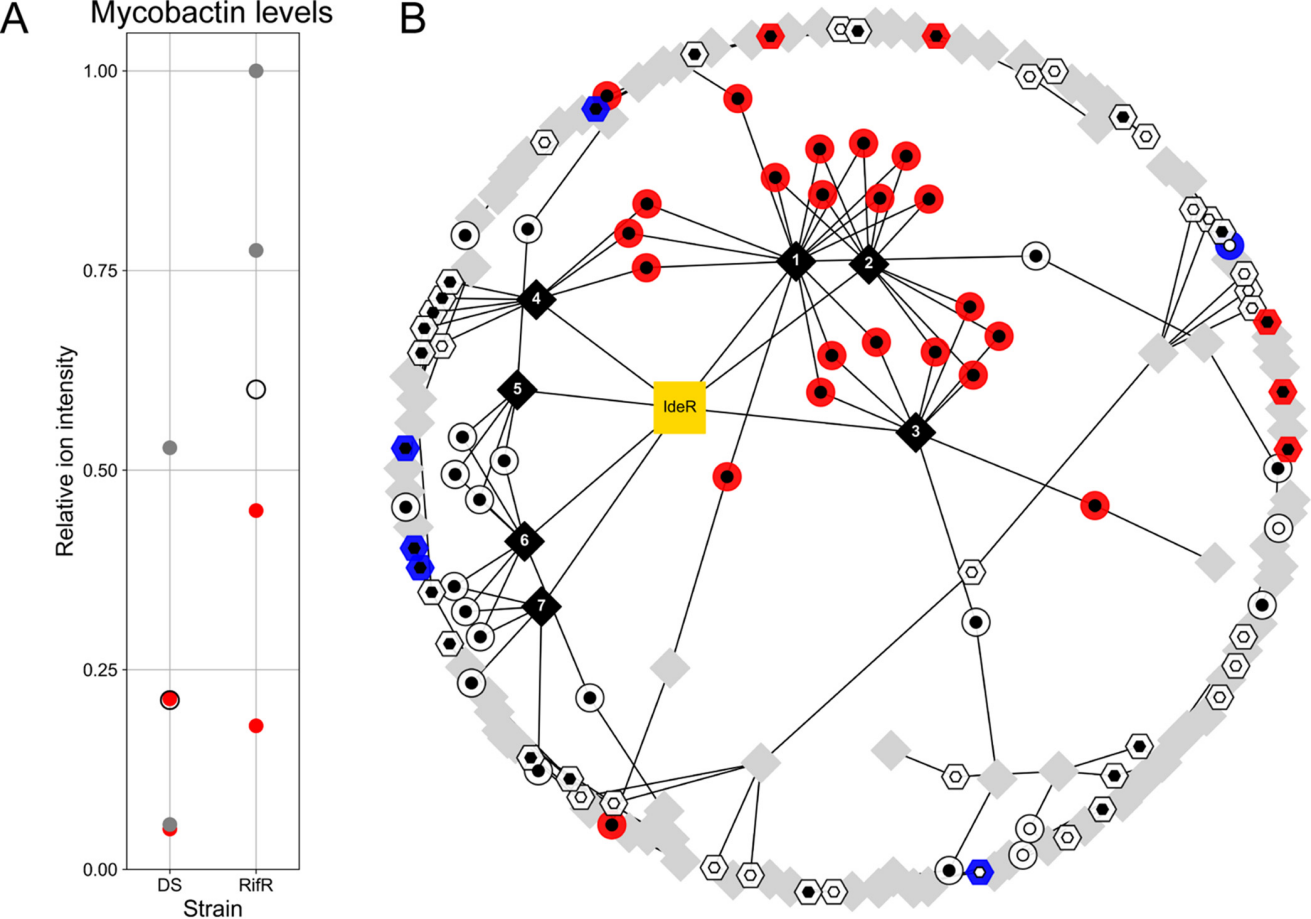
RifR had a higher baseline level of mycobactin biosynthesis than DS. (A) Relative mycobactin levels in DS and RifR in normal medium (gray dots) and iron-supplemented medium (10 μM hemin; red dots). White circles represent the mean of the observations. The highest measurement (RifR, no iron supplementation) was used as the reference value. (B) Subset of the gene regulatory network (33) containing iron-responsive genes. Circles represent IdeR-regulated genes that are either induced (black inner circle) or repressed (white inner circle) under low-iron conditions. Hexagons represent IdeR-independent iron responsive genes that are induced (white inner hexagons) or repressed (black inner hexagons) under low-iron conditions. We used blue and red to indicate significantly lower or higher RNA expression in RifR, respectively. Diamonds represent transcriptional modules as defined by Petersen et al. (33); black diamonds indicate modules that contain at least 3 IdeR-responsive genes. Edges connect gene nodes with the module nodes they belong to. Labels 1 to 7 refer to module 502 (no. 1), module 525 (no. 2), module 267 (no. 3), module 446 (no. 4), module 231 (no. 5), module 086 (no. 6), and module 295 (no. 7) from the original publication.

Next, we addressed the production of mycobactin. We prepared whole-cell extracts from DS and RifR grown in both normal medium and medium supplemented with 10 μM hemin. We found that, on average RifR, produced more mycobactin than DS (Fig. 3A), corroborating the physiological relevance of the increased baseline expression of mycobactin biosynthesis genes. We also observed a slight decrease in the production of mycobactin in bacteria grown in the hemin-supplemented medium, pointing to a modification of the expression of mycobactin biosynthesis cluster in response to iron (Fig. 3A). Given that the growth rate was not affected by the presence of hemin, these findings suggest that iron availability itself does not limit the growth rate of the mutant. It is therefore possible that the higher level of expression of the mycobactin biosynthetic cluster itself might impart a fitness cost.

Interestingly, while significantly enriched, only half of the transcripts reported to be repressed by IdeR (31) under iron-replete conditions were part of the signature of compensation (22 out of 40 genes). This prompted us to take a closer look at the IdeR regulon and its regulation. We took advantage of the availability of studies modeling the global gene regulation in M. tuberculosis (32–34). We reconstructed the genome-wide gene regulatory network and extracted the immediate neighbors of IdeR and iron-responsive genes (33). There were 7 expression modules that contained at least 3 genes that are part of the IdeR regulon (Fig. 3B, black diamonds). Together, these modules covered 82.5% of all the IdeR-repressed genes, and, with the exception of module 4 (Fig. 3B), none of the modules included IdeR-independent iron-responsive genes. All of the genes that we identified as candidates for compensation belonged to modules 1 to 4, while none of the genes included in the other modules were found to be differentially expressed in RifR. A key difference among modules was that IdeR-regulated genes represented more than half of all the genes in modules affected by compensation but fewer than half in those that were not part of the signature of compensation. Mapping proteomic data onto the same expression network produced similar results (see Fig. S6A in the supplemental material). Interestingly, few of the IdeR-independent iron-responsive genes were part of the signature of compensation. This pattern implies a modulation of the canonical function of IdeR, either through regulatory inputs from other transcription factors or via some other mechanism.

These results supported our hypothesis that mutations in *rpoB* impart changes to the baseline expression profile of M. tuberculosis that could be reversed in the presence of a compensatory mutation in *rpoC*. Combining the expression data with our findings that iron supplementation and mycobactin levels did not affect RifR growth rates, we concluded that the transcriptional changes were not driven by the demand for iron. Instead, these changes might be a reflection of a dysfunction of RNAP, e.g., differences in promoter specificity or modified interaction with IdeR, whose downstream consequences may impose a fitness effect. For example, as the mycobactin biosynthesis cluster comprises several large proteins, their excessive production could represent a drain on the cell’s resources. If true, we would expect such effects to be universal across all M. tuberculosis strains carrying this *rpoB* mutation. Indeed, mycobactin overproduction has been reported for rifampicin-resistant strains from multiple different genetic backgrounds (18).

### Mycobactin dysregulation is shaped by epistasis.

We wanted to test the hypothesis that higher expression of the mycobactin biosynthetic cluster is a general feature of rifampicin resistance in M. tuberculosis and therefore is the underlying cause of its fitness cost both in the laboratory and the clinic. To do so, we generated RpoB Ser450Leu mutants in five genetically diverse clinical isolates belonging to two different M. tuberculosis lineages and profiled them. Globally, M. tuberculosis can be grouped into nine distinct genetic lineages, each with a specific geographic distribution (35–37). M. tuberculosis lineages can differ in their interaction with the human host, the dynamics of disease progression, and also in their apparent propensity to acquire drug resistance (38, 39). We chose strains belonging to lineages 1 and 2 because of their large phylogenetic separation (see Fig. S7 in the supplemental material) and, more importantly, because drug resistance is often associated with lineage 2 and is relatively rare in lineage 1 (26). We expected that the comparison of the transcriptome and proteome between the Ser450Leu mutants and their cognate wild-type ancestor would allow us to identify general patterns of fitness cost linked to this mutation, such as mycobactin biosynthesis.

It is important to note that this comparison did not include any compensated strains, i.e., strains carrying mutations in the BBDP domain. We were therefore unable to focus our analysis exclusively on genes whose expression was corrected by the presence of an *rpoC* mutation. Nonetheless, direct comparison of RifR and DS is virtually indistinguishable from the signature of compensation when considering IdeR-regulated genes and therefore serves as a reasonable proxy for our analyses (Fig. S6B).

We started by measuring the growth characteristics of the wild-type isolates and the relative cost of the RpoB Ser450Leu mutation in the different strain backgrounds. The generation time varied from 22.7 h (95% CI, 20.8 to 25.0 h) to 31.0 h (95% CI, 29.3 to 35.1 h). The relative fitness cost of the RpoB Ser450Leu mutation differed as well, from a modest 2% (mixed-effects linear regression; *P* = 0.71) to a pronounced 27% (mixed-effects linear regression; *P* = 5.6 × 10^−6^) (see Table S1 in the supplemental material).

**FIG 4:**
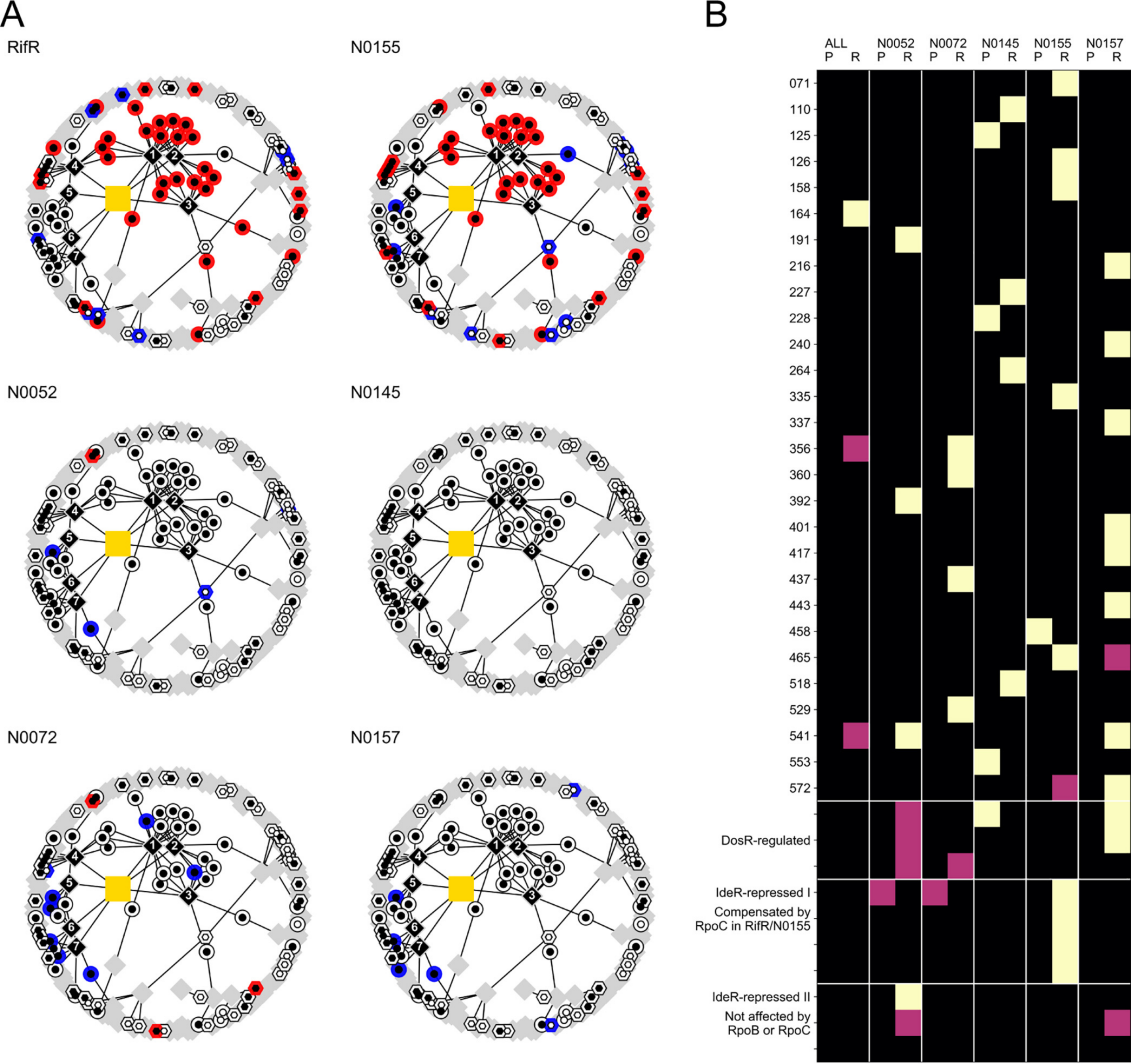
The prominent role of mycobactin biosynthesis in the signature of compensation was not universal. (A) Iron-responsive subset the of gene regulatory network, as shown in Fig. 3, colored based on transcriptional differential expression data from pairwise comparison of genetically distinct rifampicin-susceptible clinical isolates and their cognate RpoB Ser450Leu mutants. RifR and N0155 refer to an independent sampling of the same strain pair. See Fig. S7 in the supplemental material for the proteome counterpart of this plot. (B) Representation of the enrichment of significantly differentially expressed genes within individual transcriptional modules, as defined by Peterson et al. (33). The columns alternate proteomic (P) and transcriptomic data (R). “ALL” refers to the global differential expression analysis of all rifampicin-susceptible against all rifampicin-resistant strains. The remaining column annotations refer to individual pairwise comparisons in different genetic backgrounds. Black squares represent no significant enrichment, and mauve squares and yellow squares show enrichment at 0.01 < *P* < 0.05 and *P* < 0.01 using Fisher’s exact test. These *P* values are not adjusted for multiple testing. Modules covering the DosR regulon and IdeR iron-repressed regulon are highlighted separately.

We obtained the expression profiles for each strain to check whether the pattern we identified for IdeR-repressed genes was a universal phenotype for RpoB Ser450Leu mutants. When we analyzed the transcriptomic data by performing a single comparison across the five strain pairs, we found that only 17.5% (7/40) of the IdeR-repressed genes were significantly differentially expressed. A single gene belonging to the mycobactin biosynthesis cluster was included in that number. Proteomic analysis revealed a similar result; 17.1% (6/35) of detected proteins, none of which belonged to the mycobactin biosynthesis cluster, were found to be significantly differentially expressed across all strains. None of the iron homeostasis gene sets highlighted in the signature of compensation were significantly differentially expressed across all strains. Since these findings were contrary to our expectations, we stratified the analysis and mapped the differential expression results for each strain onto the IdeR and iron-responsive gene network we collated earlier. These results echoed our combined analysis, namely, the signature of compensation was not universal across the tested strains. N0155, which corresponds to DS, is the only strain to show a transcriptional profile consistent with the signature of compensation (Fig. 4). Proteomic data corroborated this finding (see Fig. S8 in the supplemental material). It is important to note that these data represent an independent replication of the experiments from which we derived the signature of compensation, showing that our original results are robust and reproducible. However, the absence of a coherent IdeR-responsive phenotype in the other strains was clear evidence that mycobactin dysregulation is not a universal feature of rifampicin resistance, but rather is shaped by the genetic makeup of each strain—in other words, epistasis. The fact that mycobactin dysregulation was not generalizable raised a broader question: are there any commonalities in the phenotypic manifestation of the RpoB Ser450Leu mutation among our set of strains?

### Correlates of rifampicin resistance-related fitness cost.

Our profiling of RifR (N0155) provided compelling evidence that the dysregulation of gene expression mediated the cost of rifampicin resistance. The data generated to expand on this observation, while not supportive of a general role of mycobactin dysregulation, indicated that fitness costs differ across genetic backgrounds. We reasoned that this gradient could be exploited to identify correlates of rifampicin resistance-related fitness cost and therefore to generate new hypotheses to be explored in the future. Before we could proceed, we needed to account for the possibility that expression differences might be driven by the genetic relatedness of strains, therefore limiting our inferences about the impact of RpoB Ser450Leu. To do so, we performed a pairwise comparison based on genetic and expression relatedness (see supplemental material). We found that the impact of resistance on the expression profile of any two strains was independent of the genetic distance between them (see Fig. S9 in the supplemental material). We surmised that the specific phenotypic manifestation of resistance was therefore dependent on genetic variation that defined strains rather than lineages. As a result, our data could be used to find correlates of the differing fitness costs of RpoB Ser450Leu.

**FIG 5:**
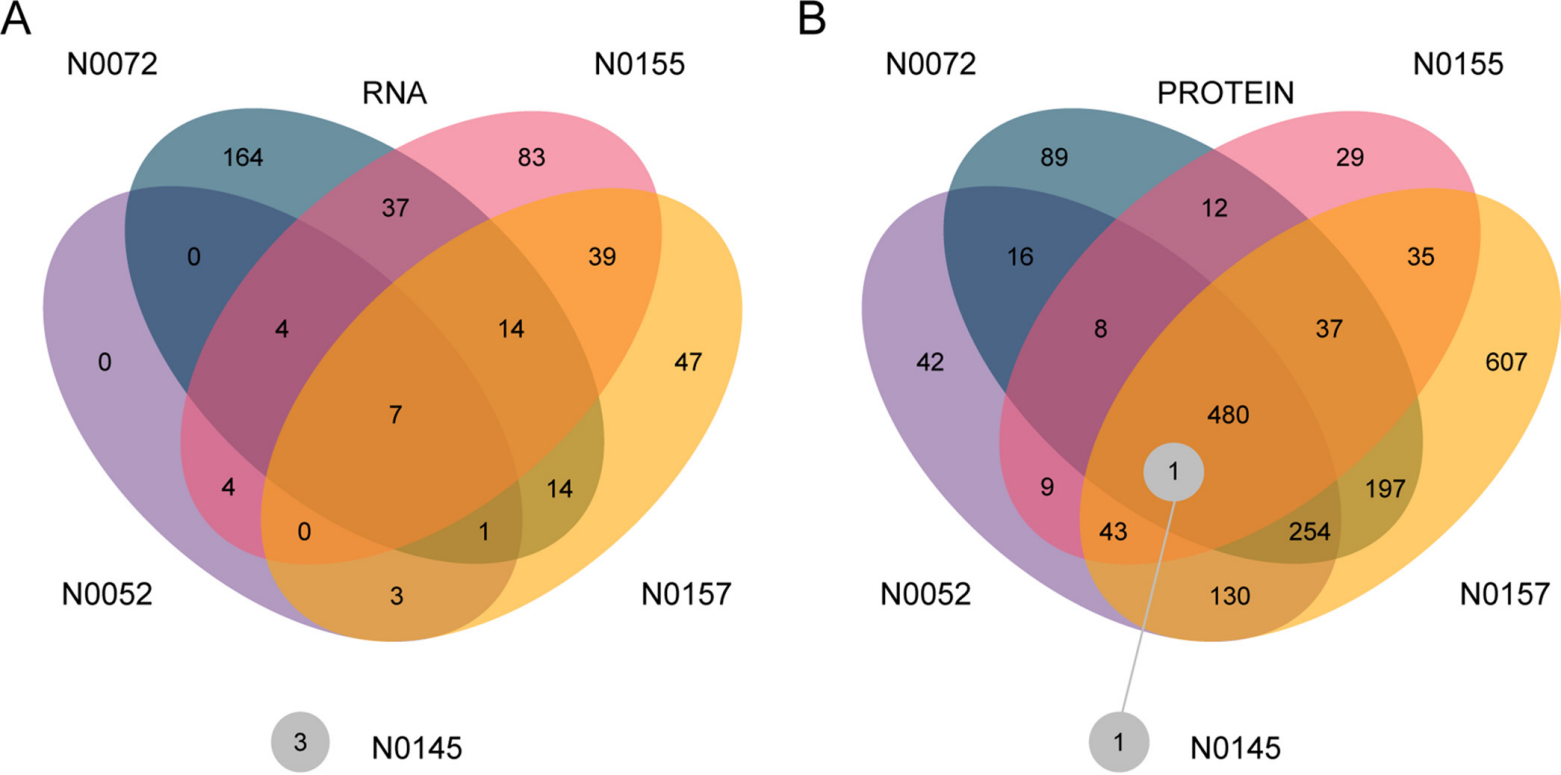
The impact of RpoB Ser450Leu on gene expression varied across strains and cellular compartments. (A) Venn diagram of differentially expressed transcripts (*P* < 0.05 after adjustment for multiple testing; RNAseq) between clinical strains and their cognate RpoB Ser450Leu mutant. (B) Venn diagram of differentially expressed proteins (*P* < 0.05 after adjustment for multiple testing; SWATH-MS) between clinical strains and their cognate RpoB Ser450Leu mutant.

Overall, we were able to detect a wealth of gene expression changes in our samples; as many as 958 transcripts and 1,914 proteins were observed to be differentially expressed in at least one comparison across our samples. The effect on individual strains differed, ranging from a few genes for N0145 to almost half of the genome in N0157. Our attempt to identify common patterns resulted in two contrasting lines of evidence. On the transcriptional level, each strain was perturbed in its own private way (Fig. 5A), manifesting as the drug resistance iteration of the Anna Karenina principle (40). Of the 958 transcriptional changes, none affected all five tested strain pairs, and only seven were shared by four strain pairs. This heterogeneity was also supported by analyses focused on transcriptional modules and the comparison of the magnitude of differential expression. While we were able to identify individual transcriptional modules as dysregulated in individual backgrounds, there were no commonalities across strains (Fig. 4B). Furthermore, we saw almost no association between the relative fitness of RpoB Ser450Leu mutants and the magnitude of global transcriptional disruption (*R*^2^ = 0.39; *P* = 0.258; ordinary least-squares linear regression; see Fig. S10 in the supplemental material). The fact that the same mutation, RpoB Ser450Leu, can have such profoundly different outcomes depending on the genetic context in which it occurs is further evidence of the epistasis we observed in the regulation of mycobactin described above. It also shows that natural genetic variation across strains can fundamentally impact the physiological consequences, and by extension, the evolution of drug resistance in M. tuberculosis.

In contrast to the transcriptional changes, the pattern of proteome disruption was more coherent across strains. While there was clear evidence for idiosyncratic strain-dependent differences, with only one shared differentially expressed protein across all strain pairs, the level of 480 proteins (17% of the measured proteins) was significantly altered in four rifampicin-resistant strains compared those in to their ancestors (Fig. 5B). This overlap points to a more consistent impact of RpoB Ser450Leu than that suggested by the transcriptome and hints at a broader posttranscriptional readjustment of expression. Moreover, we found a correlation between the relative fitness cost of the RpoB mutation and the extent of proteome disruption caused by this mutation in the different backgrounds (*R*^2^ = 0.86; *P* = 0.022; ordinary least-squares linear regression; see Fig. S10).

We used our proteomic data to identify which metabolic pathways were most affected by a change in the relative abundance of their constitutive proteins. We found that RpoB Ser450Leu imparted a broad recalibration of central carbon metabolism, with an upregulation of proteins involved in glycolysis/gluconeogenesis, the citric acid cycle, and amino acid metabolisms (see Fig. S11 and supplemental note in the supplemental material). Interestingly, the majority of the differentially expressed proteins showed an increase in baseline levels of protein abundance, hinting at a greater investment in the protein compartment. Indeed, accounting for the amino acid composition and size of individual proteins suggested that the proteome of *rpoB* mutants seemed to require a larger investment of biomass than their wild-type ancestors (see Fig. S12 in the supplemental material).

Whether these changes are the cause or consequence of the fitness cost of RpoB Ser450Leu remains to be determined. Nonetheless, the study of growth rate-dependent gene expression by itself, and of how it pertains to RNA polymerase mutations, might point to new physiological vulnerabilities of rifampicin-resistant M. tuberculosis.

## DISCUSSION

**FIG 6:**
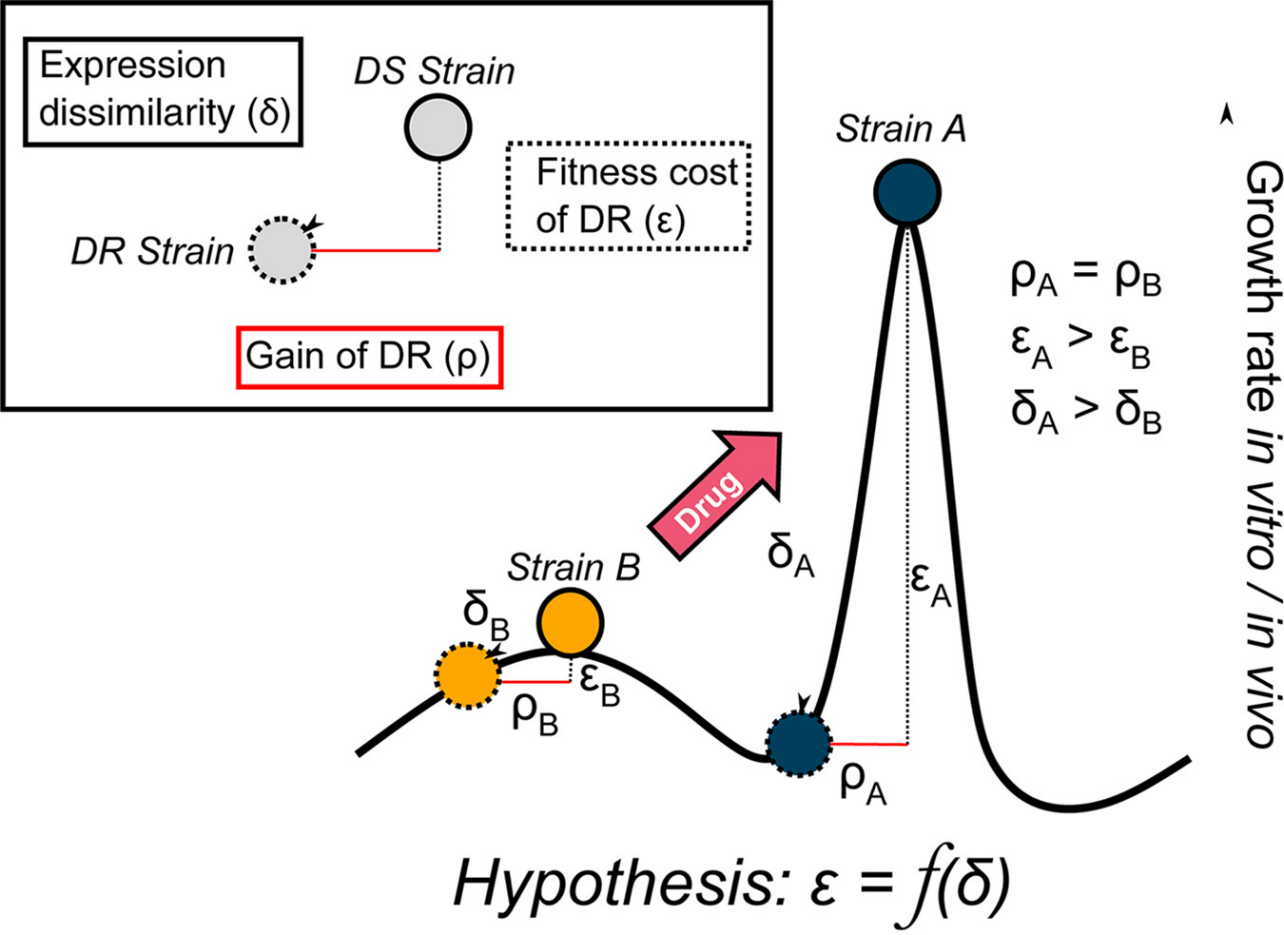
Fitness cost of rifampicin resistance as a function of the dysregulation of gene expression. Based on our observations, we propose that the gain of a rifampicin-resistance conferring mutation (ρ) turns a drug-susceptible (DS) strain into a drug-resistant (DR) strain. However, it also imparts a global change in gene expression (δ). The extent of this change determines the fitness cost (ε) of the rifampicin resistance-conferring mutation. Consider the fitness landscape comprising two strains (strain A and strain B). According to our hypothesis, if the acquisition of the same rifampicin resistance-conferring mutation in both strains (ρ_A_ = ρ_B_) imparts a larger disruption of gene expression in strain A than in strain B (δ_A_ > δ_B_), we would expect a greater loss of reproductive fitness in strain A than in strain B (ε_A_ > ε_B_). Consequently, therapeutic agents (“drug”) that would result in the *de facto* movement of strain B across the fitness landscape could increase the fitness cost of drug resistance in strain B.

We show that RpoB Ser450Leu imparts a measurable physiological perturbation in addition to conferring rifampicin resistance. Consistent with the suggested role of compensatory mutation (10, 14, 15, 27), we confirmed that in one strain, RpoC Leu516Pro reduced both the apparent fitness cost of rifampicin resistance, defined as reduced growth rate in the absence of drug, and the magnitude of the expression changes arising from it. However, we also found that the nature of the perturbation was not consistent across different genetic backgrounds. Higher expression of the mycobactin biosynthetic cluster correlated with the cost of rifampicin resistance in one clinical strain, but remained unperturbed in the other four unrelated strains. Instead, we observed a strain-specific response to the RpoB mutation, in terms of both the relative impact on growth and the rearrangement of gene expression. A general mechanism of fitness cost for even a single rifampicin resistance-conferring mutation remains elusive and is subject to modulation by the particular genomic context. Using the data presented in this study, we propose a hypothesis for how the gain-of-resistance-conferring mutation, changes in gene expression, and relative fitness of rifampicin-resistant mutants might operate (Fig. 6).

One of our main assertions emerging from this work is that the fitness cost of rifampicin resistance and its compensation are mediated by expression differences. This claim is based on the fact that the presence of the compensatory Leu516Pro substitution in RpoC partially restores many of the expression changes observed in RifR while reducing the cost of drug resistance. Among these, dysregulation of mycobactin biosynthesis provided the strongest reproducible functional signal, affecting both transcripts and proteins. Iron homeostasis is essential for optimal growth, both in culture and during infection, and its disruption could impair the ability of M. tuberculosis to proliferate and cause disease. Expression of the mycobactin cluster is repressed under iron-replete conditions by IdeR (31), and its upregulation in RifR indicates insufficient access to iron. Nonetheless, we found no association between iron supplementation and the growth rate of RifR. Furthermore, we observed incoherent expression of the IdeR regulon, which indicates aberrant expression rather than a physiologically accurate response to iron. Together, these observations point to the expression of the mycobactin biosynthetic cluster, rather than insufficient iron, as a most likely cause for slower growth. However, it is possible that the growth rate differences were mediated by a different signal—either the modified expression of a different protein, or group of proteins or, alternatively, a small-molecule signal such as cAMP (41). While we cannot exclude this possibility, it is unlikely that other coherent expression differences account for the impact of resistance, as illustrated by the disparity in expression profiles among strains. An important caveat to this is that our “signature of compensation” was based on a single strain background. Ideally, we would validate our findings in a separate “isogenic” strain set; however, we did not have access to such strains. We attempted to address the issue of generalizability by analyzing additional *rpoB* mutants from different genetic backgrounds. Similarly, we only explored the effects of a single—albeit the clinically most relevant—RpoB mutation, Ser450Leu. While this choice was intentional to control for the disparate consequences that different mutations might have on RNAP (8, 15, 42), our observations may not hold true for other RpoB mutations. Finally, we did not attempt to quantify *in vivo* transcriptional efficiency using reporter systems (8, 9). Instead, we assumed that the transcriptional properties determined *in vitro* (15) were applicable to and comparable in different strains. An indirect line of evidence supporting the validity of this assumption is provided by the fact that RNAP and ribosomal protein levels, both linked to transcriptional efficiency (8, 43, 44), were mostly unaltered among the strains we analyzed (see Fig. S13 and S14 in the supplemental material).

Keeping these considerations in mind, there are two striking features to emerge from our results. The first is the pervasive epistasis modulating the impact of RpoB Ser450Leu; the same mutation had markedly different effects on the physiology of different M. tuberculosis strains. The second is the apparent mechanism through which modulation of gene expression is propagated across the levels of bacterial physiology. Modification in RNAP function seems to have pleiotropic effects that transcend the disruption of any single group of genes and impart instead a global perturbation of gene expression.

One question that remains open is that of what sits at the heart of the disparity in phenotypes. The sequence of RNAP is effectively the same in all strains (26) and, by extension, so should be all the biochemical changes that arise from resistance. Namely, RNA polymerase with a Ser450Leu substitution exhibits lower transcriptional initiation efficiency, lower elongation rates, and a higher efficiency of transcriptional termination (15). All of these phenotypes are mitigated by the presence of mutations in the BBDP domain in RpoC (15). If these defects are intrinsic to the enzyme and therefore should have a coherent impact, what gives rise to the observed phenotype heterogeneity? We envisage that part of the answer lays in differences in underlying robustness—a strain’s capacity to buffer perturbation. One possible candidate for this is DnaJ2, a chaperone that was recently reported to be important for the stabilization of the Ser450Leu substitution in RpoB of mycobacteria (45). For example, differing physiological levels of this protein could provide a rationale for our observations. Unfortunately, we did not find a statistically significant correlation between DnaJ2 levels and the relative fitness of RpoB mutants (see Fig. S15 in the supplemental material). On the other hand, we can consider our data set a window into the evolutionary adaptation of each strain, and a sign of how different their physiologies really are. The amalgamation of mutational differences that effectively makes up a strain’s genetic background weaves a baseline phenotype that allow different M. tuberculosis strains to be successful pathogens despite differences in their underlying physiology (46). These differences are unmasked by the presence of a mutation that sits at the core of gene expression and reveals idiosyncratic transcriptional responses to rifampicin resistance that are poorly conserved across phylogenetic distances. This observation implies that further investigation of positive selection of compensation of resistance-related traits should be performed in genetically very closely related strains, as results could differ considerably when comparing across larger phylogenetic distances. In the case of our study, even two relatively similar strains (N0145 and N0155, 207 single-nucleotide polymorphisms apart) were found to have considerably different physiological consequences of the same mutation. Indeed, most studies that attempted to identify signatures of drug resistance-driven positive selection of traits have generated diffuse signals (47–49).

The strain-specific nature of resistance-related expression perturbations provides a tractable link to disparate growth rate modulation. There is a growing body of literature exploring how proteome composition influences growth rate (50–52). Such studies have led to the formulation of a collection of “growth laws” that link growth rates to the partitioning of the proteome between ribosomes and the proteins carrying out other cellular functions (51). Growth on different carbon sources impacts this balance, with “poorer” carbon sources requiring a greater investment into the functional proteome, presumably because of the need for anabolic reactions that increase the reliance on biosynthetic enzymes. A similar relationship has been observed in a wide range of microbial species (53). An elaboration of these growth relationships also led to the conclusion that the efficiency of proteome allocation can impact growth rates and cell physiology (54). Our finding that the increase in the biomass investment into the proteome brought about by the gain of an RpoB mutation correlates with the relative fitness of that mutation is consistent with these reports. However, before we can claim that proteome allocation drives growth rate in M. tuberculosis in a way that is analogous to that in E. coli, we will first have to establish what limits the growth rate of an M. tuberculosis cell. In E. coli, proteins make up at least half of the dry weight of the cell (51), while in M. tuberculosis this fraction is considerably lower, around 20% (55). While the relationship is not impossible, there are other components that represent sizable investment into biomass in M. tuberculosis. For example, both lipids and cell wall may act as a sink for growth-limiting resources in M. tuberculosis, as they can account for more than half of the dry mass of actively growing cells (55). As mentioned earlier, lipidomic analysis pointed to differences in mycobactin biosynthesis as one of the biggest discrepancies between rifampicin-resistant mutants and their susceptible ancestors (18). While echoing a key observation from our quest for determining the cost of resistance, we saw no evidence that mycobactin levels themselves change the rate of bacterial growth. The virulence-associated phthiocerol dimycocerosates (PDIM) have also been implicated in the cost of rifampicin resistance (19), as have other changes in lipid composition (20). The full exploration of the role of lipids in the physiology of rifampicin-resistant M. tuberculosis is beyond the scope of this study. Nonetheless, it would provide an interesting new and complementary avenue to pursue, especially when correlated with the expression of the pertinent biosynthetic proteins. Moreover, the physiological aspects of growth rate determination in M. tuberculosis remain poorly defined, and such studies could provide important insights.

In conclusion, the observed differential cost of rifampicin resistance across M. tuberculosis strains provides a lens through which we can better understand the emergence of drug resistance in clinical tuberculosis. Such considerations illuminate a new avenue to pursue in the fight against rifampicin-resistant M. tuberculosis and perhaps to uncover a new paradigm for chemotherapeutic intervention. If the disruption of proteome composition indeed disproportionally impacts *rpoB* mutants, then agents that impart a considerable shock to the expression equilibrium of bacteria could exhibit potent activity against rifampicin-resistant strains due to collateral sensitivity (56)—provided, of course that the strains have not yet been compensated. For example, the caseinolytic protease and the proteasome both play an essential role in protein homeostasis in M. tuberculosis (57, 58) and, as such, represent interesting intervention points. Indeed, both have garnered considerable attention as underexplored antibiotic targets (58). As a consequence, when given in combination with rifampicin, such agents may act to suppress the emergence of resistance by imposing a physiological barrier, a valuable attribute for lengthening the shelf life of rifampicin. Exploring the causality of the relationship between proteome disruption and the cost of rifampicin resistance, and the impact of growth rate on M. tuberculosis gene expression, could provide a framework for enabling collateral sensitivity approaches in M. tuberculosis (56).

## MATERIALS AND METHODS

### Strains and culture conditions.

We used four strains described previously (27), namely, the wild type, clinical isolate T85 (N0155 [DS]), a rifampicin-resistant mutant of T85 carrying the Ser450Leu mutation (N1981 [RifR]), a derivative of T85 that was evolved by serial passage (200 generations) in the absence of rifampicin (N1588 [DSevo]), and an evolved derivative of the rifampicin-resistant strains carrying an additional mutation in RpoC, Leu516Pro (N1589 [RifRevo]).

In addition to these strains, we used four clinical isolates that are part of a reference set of M. tuberculosis clinical strains covering the genetic diversity of M. tuberculosis (26). Two strains belonged to lineage 1 (N0072 and N0157) and two to lineage 2 (N0052 and N0145). We plated each of these strains on 7H10 plates containing 5 μg/ml rifampicin and picked colonies of spontaneous mutants. We checked the rifampicin resistance-conferring mutations using Sanger sequencing of the amplified RRDR region (forward primer, TCGGCGAGCTGATCCAAAACCA; reverse primer, ACGTCCATGTAGTCCACCTCAG; product size, 601 bp), and kept a Ser450Leu derivative of each respective clinical strain (N2027, N2030, N2495, and N1888).

Bacteria were cultured in 1-liter bottles containing large glass beads to avoid clumping and 100 ml of medium, incubated at 37°C and rotated continuously on a roller. Unless otherwise, stated we used a modified 7H9 medium supplemented with 0.5% wt/vol pyruvate, 0.05% vol/vol tyloxapol, 0.2% wt/vol glucose, 0.5% bovine serum albumin (Fraction V; Roche), and 14.5 mM NaCl. Compared to the usual composition of 7H9, we omitted glycerol, Tween 80, oleic acid, and catalase from the medium. We added 10 μM hemin (Sigma) when supplementing growth medium with iron. We followed growth by measuring optical density at 600 nm (OD_600_).

### Data analysis.

Unless otherwise stated, we preformed the analyses using Python 3.5.2 augmented with the following modules to provide additional functionality: Matplotlib (v2.0.0), Numpy (v1.12.1), Scipy (v0.19.0), Pandas (v0.20.1), statsmodels (v0.8.0), sklearn (v0.18.1), and netwrokX (v2.5.1).

### Fitness determination.

M. tuberculosis fitness was determined by comparative growth rate estimation. We grew bacteria as described and followed their growth by measuring OD_600_. We transformed the optical density measurements using logarithm base 2 and trimmed all early and late data points that deviated from the linear correlation expected for exponential growth. Next, we fitted a linear mixed-effects regression model to the data. Fitness cost was calculated as the resistance-imposed deviation from wild-type growth dynamics.

### Transcriptional analysis with RNAseq.

We transferred a 40-ml aliquot of bacterial culture in the mid-log phase (OD_600_ = 0.5 ± 0.1) into a 50-ml Falcon conical tube containing 10 ml ice. We harvested the cells by centrifugation (3,000 × *g*, 7 min, 4°C), resuspended the pellet in 1 ml of RNApro solution (MP Biomedicals), and transferred the suspension to a lysing matrix B tube (MP Biomedicals). We disrupted the bacterial cells using a FastPrep24 homogenizer (40 s, intensity setting of 6.0; MP Biomedicals). We clarified the lysate by centrifugation (12,000 × *g*, 5 min, 4°C), transferred the supernatant to a clean tube and added chloroform. We separated the phases by centrifugation (12,000 × *g*, 5 min, 4°C) and precipitated the nucleic acids from the aqueous phase by adding ethanol and incubating at −20C overnight. We performed a second acid-phenol extraction to enrich for RNA. We treated our samples with DNase I Turbo (Ambion), and removed stable RNAs by using the RiboZero Gram-positive rRNA depletion kit (Epicentre). We prepared the sequencing libraries using the TruSeq stranded total RNA kit (Illumina) and sequenced on a HiSeq 2500 high-output run (50 cycles, single end).

Illumina short reads were mapped to the M. tuberculosis H37Rv reference genome using Burrows-Wheeler Aligner (BWA; v0.7.13); the resulting mapping files were processed with SAMtools (v1.3.1). Per-feature read counts were performed using the Python module htseq-count (v0.6.1p1) and Python (v2.7.11). We performed differential expression analysis using the R package DESeq2 (v1.16.1) (59) and R (v3.4.0). In the case of the identification of the signature of compensation we performed a comparison of RifR versus DS plus DSevo plus RifRevo. For the follow-up experiments, we performed two separate comparisons, (DR_N0072_ + DR_N0157_ + DR_N0052_ + DR_N0145_ + DR_N0155_) versus (DS_N0072_ + DS_N0157_ + DS_N0052_ + DS_N0145_ + DS_N0155_), as well as individual DR versus DS comparisons for each strain.

Gene set enrichment analysis was based on functional annotation from the Kyoto Encyclopedia of Genes and Genomes (KEGG) and a custom collation of curated gene sets based on published reports. The overrepresentation analysis was based on Fisher’s exact test as the discriminating test.

In addition, we transformed per-feature counts into transcript counts per million bases (TPM). TPM for each feature for each sample were calculated using the following formula:

$${\text{TPM}}_{i}=\frac{\frac{{\text{counts}}_{i}}{{\text{size}}_{i}}}{\sum_{j}^{n}\frac{{\text{counts}}_{j}}{{\text{size}}_{j}}}$$

where counts*_i_* refers to the number of reads that map to a feature *i* and size*_i_* refers to the length (in bp) of feature *i*. This ratio was normalized by dividing by the sum of all the ratios across all the features.

### Proteomic analysis with SWATH-MS.

We harvested 20 OD_600_ equivalents from mid-log phase (OD_600_ = 0.5 ± 0.1) bacterial cultures by centrifugation (3,000 × *g*, 7 min, 4°C). We washed the bacterial pellet twice with phosphate-buffered saline (PBS) to remove residues of tyloxapol. We resuspended the bacterial pellet in 500 μl of protein lysis buffer (8 M urea, 0.1 M ammonium bicarbonate, and 0.1% RapiGest; Waters) and transferred the suspension to a lysing matrix B tube (MP Biomedicals). We disrupted the bacterial cells using a FastPrep24 homogenizer (40 s, intensity setting of 6.0; MP Biomedicals). We clarified the lysate by centrifugation (12,000 × *g*, 5 min, 4°C), and sterilized the supernatant by passing it twice through 0.22-μm syringe filters (Millipore).

Following protein extraction for each sample, we used trypsin to digest proteins into peptides and then desalted them using C_18_ columns (The Nest Group). The cleaned-up peptides were resuspended in mass spectrometry buffer (2% [vol/vol] acetonitrile and 0.1% [vol/vol] formic acid). Finally, index Retention Time (iRT) kit (Biognosis) containing 11 iRT retention time normalization peptides was spiked into every sample.

We measured every sample in sequential window acquisition of all theoretical mass spectra (SWATH) mode, a data-independent acquisition implementation, on a TripleTOF 5600 mass spectrometer (AB Sciex) coupled to a nanoflow high-performance liquid chromatography (HPLC) system with a gradient of 1 h (60). The raw files acquired through a 64 variable-width-window precursor isolation scheme were centroid normalized using ProteoWizard msconvert. We used a M. tuberculosis spectral library described previously (61) to extract data using the OpenSWATH workflow (60, 62, 63). The processed data were filtered by MAYU to 1% protein false-discovery rate (FDR) (63). The R packages aLFQ and MSstats were used for protein quantification (top 3 peptides and top 5 fragment ions [64]) and differential expression analysis, respectively (65, 66).

### Mycobactin determination.

We harvested 5 OD_600_ equivalents from mid-log phase (OD_600_ = 0.5 ± 0.1) bacterial cultures by centrifugation (3,000 × *g*, 7 min, 4°C). We washed the bacterial pellet three times with 15 ml of cold, sterile 7H9 medium base devoid of additives (BD) to remove residues of tyloxapol. After washing, we resuspended the pellets in 80 μl of cold, sterile 7H9 medium base and added 750 μl of 1:2 chloroform-methanol. We vortexed the samples for 5 min at top speed and added 750 μl of chloroform. The samples were shaken for 1.5 h at room temperature and clarified by centrifugation (16,000 × *g*, 10 min). We transferred the organic phase to a fresh tube, dried the samples in a SpeedVac vacuum concentrator, and resuspended each sample in 120 μl of 44:44:2 (vol/vol/vol) acetonitrile-methanol-H_2_O.

Chromatographic separation and analysis by mass spectrometry was done using a 1200 series HPLC system with a Kinetex column (1.7 μl × 100 mm × 2.1 mm; Phenomenex) with a SecurityGuard Ultra column guard (part no. AJ-9000) coupled to a 6550 accurate-mass Q-TOF instrument (Agilent Technologies). Solvent A consisted of H_2_O and 10 mM ammonium acetate; solvent B consisted of acetonitrile and 10 mM ammonium acetate. A 10-μl aliquot of extract was injected, and the column (C_18_) was eluted at 1.125 ml/min. Initial conditions were 60% solvent B, followed by 0 to 2 min at 95% B, 2 to 4 min at 60% B, and 4 to 5 min at initial conditions. Spectra were collected in negative-ion mode from 50 to 3,200 *m*/*z*. Continuous infusion of calibrants (compounds HP-321, HP-921, and HP-1821; Agilent) ensured exact masses over the whole mass range.

We converted the raw data files to the mzML format using msConvert and processed them in R using XCMS (67) (v3.0.2). We extracted targeted ion chromatograms with CAMERA (v1.34.0).

### Transcriptional module analysis.

The iron-responsive subgraph of the global gene regulation network published by Peterson et al. (33), was generated by using all expression modules and all iron-responsive genes as nodes, with edges connecting them representing module membership. All other gene nodes were discarded, keeping only the information pertinent to the number of genes present in each module (its degree). We focused explicitly on modules with at least 3 IdeR-dependent iron-responsive genes within them. Finally, we marked significant differential expression of the gene nodes in every comparison.

For the purposes of contextualizing the expressional profiling of RpoB Ser450Leu, we selected a subset of expression modules as follows. First, we collated all of the genes that were differentially expressed in at least one genetic background, as determined by pairwise comparisons. We then scored each expression module for enrichment of membership by differentially expressed genes using a binomial test. We retained all modules for which the test pointed to an excess of differentially regulated genes (*P* < 0.05). We constructed a new subgraph of the global regulatory network using all enriched modules and their constituent genes, irrespective of whether or not individual genes were significantly differentially expressed. Edges reflected module membership. We added expression information in the form of log fold changes of abundance to each subgraph based on pairwise analyses.

### Calculation of genetic distance between clinical isolates.

Genetic distance between strains was defined as the number of single-nucleotide variants (SNV) that separate two strains. The numeric value of this parameter was extracted from a phylogeny published elsewhere (26).

### Quantification of the relative impact of the *rpoB* mutation on gene expression in different clinical isolates.

We define the dissimilarity in the expressional response to the presence of the *rpoB* mutation using the following three metrics: the absolute number of shared significantly differentially expressed genes, the fraction of both the shared significantly differentially expressed genes and shared nonaffected genes (Hamming distance), and the Euclidean distance between ratios of TPM. The first is simply the number of shared genes that were found to be significantly affected by the presence of the *rpoB* mutation in two different genetic backgrounds. For the second, we used the same input to calculate the Hamming distance between the patterns of genes significantly affected by the mutation in *rpoB* in two different genetic backgrounds. In the third case, we first calculated the TPM. We then calculated the mean TPM for each gene across the biological replicates, as well as the ratio of mutant to wild-type mean TPM for every gene. This gave us a vector containing 4,000 ratios for each mutant-wild type pair. Finally, we calculated the Euclidean distance between these vectors for the different genetic backgrounds. We plotted each of these metrics against genetic distance and calculated the Spearman correlation and the coefficient of variance as follows: standard deviation over mean multiplied by 100 (σ/μ × 100%).

### Quantification of the degree of compensation from global expression data.

We first extracted the log fold change for each gene calculated with DESeq2 and MSstats in the comparison between either DS-RifR or RirR-RifRevo. We then performed an ordinary least-squares linear regression either on the complete complement of quantified genes for each compartment (RNA or protein) or exclusively on genes that we consider to be part of the signature of compensation. The assumption of this analysis was that the slope should fall in the range between 0 (no compensation) and 1 (perfect compensation). Furthermore, the slope estimated from fitting to all quantified genes provides an estimate of the overall degree of compensation, while focusing only on the signature of compensation provides us with an estimate for the genes that we deem to be more relevant to the fitness cost of rifampicin resistance-conferring mutations.

### Proteome allocation changes.

We determined the cumulative change in the abundance of proteins belonging to a metabolic pathway by using the label-free quantification (LFQ) data derived from SWATH-MS and combining them using metabolic pathways defined by the Kyoto Encyclopedia of Genes and Genomes. We first calculated the mean proportion (μ) of the total proteome (*P*) that a given protein (*p_i_*) represented in strain *j* across *N* samples (*j_1_* to *j_N_*), as follows:

$$\mu_{p_{i,j}=}\frac{\sum_{j=1}^{N}\frac{p_{i,j}^{\text{LFQ}}}{\sum_{n=1}^{k}P_{n,j}^{\text{LFQ}}}}{N}$$

We then calculated the mean difference in the protein allocation (δ) for protein *i* across the *X* different tested M. tuberculosis genotypes (*j*_1_ to *j_X_*) when comparing the different wild-type (WT) strains to their cognate RpoBSer450Leu using the formula below.

$$\delta_{i}=\frac{\sum_{j=1}^{X}(\mu_{p_{i,j}}^{\text{WT}}-\mu_{p_{i,j}}^{{\text{RpoB}}^{\text{Ser450Leu}}})}{X}$$

Finally, we calculated the difference in the fractional allocation to a metabolic pathway (C) by summing all the mean differences in the protein allocation for each protein (*i*, 1 to *k*) in that pathway as defined by KEGG.

$$\Delta_{C}=\overset{k}{\underset{i=1}{\sum }}\delta_{i}$$

The results of this analysis were visualized using FuncTree (https://bioviz.tokyo/functree/).

### Data availability.

All RNAseq data were deposited in the ArrayExpress repository of the European Bioinformatics Institute under accession no. E-MTAB-7359. The mass spectrometry proteomics data have been deposited to the ProteomeXchange Consortium via the PRIDE (68) partner repository with the data set identifier PXD011568. Processed data and the record of the analysis were deposited on Zenodo (http://dx.doi.org/10.5281/zenodo.4903635) and GitHub as Jupyter notebooks (https://github.com/SwissTPH/TBRU_RIFcost).
