# Supplementary material for: Expression Dysregulation as a Mediator of Fitness Costs in Antibiotic Resistance
Source: Antimicrob Agents Chemother. 2021 Aug 17;65(9):e00504-21. doi: 10.1128/AAC.00504-21 (PMC8370218; doi:10.1128/AAC.00504-21)
Supplement: Supplemental file 1 — Supplemental material. Download AAC.00504-21-s0001.pdf, PDF file, 1.0 MB [file aac.00504-21-s0001.pdf]

Expression dysregulation as a mediator of fitness costs in antibiotic resistance.

Supplementary Material

Andrej Trauner[1,2], Amir Banaei-Esfahani[3,4], Sebastian Gygli[1,2], Philipp Warmer[3], Julia Feldmann[1,2], Mattia Zampieri[3], Sonia Borrell[1,2], Ben C. Collins[3], Christian Beisel[5], Ruedi Aebersold[3,6], and Sebastien Gagneux *[1,2]

[1]Department of Medical Parasitology and Infection Biology, Swiss Tropical and Public Health Institute, Basel, Switzerland
[2]University of Basel, Basel, Switzerland
[3]Department of Biology, Institute of Molecular and Systems Biology, ETH Zurich, Zurich, Switzerland
[4]PhD Program in Systems Biology, Life Science Zurich Graduate School, University of Zurich and ETH Zurich, Zurich, Switzerland
[5]Genomics Facility Basel, Department of Biosystems Science and Engineering, ETH Zurich, Basel, Switzerland
[6]Faculty of Science, University of Zurich, Zurich, Switzerland

*sebastien.gagneux@swisstph.ch

List of Figures

1 Relationship between bacterial fitness, measured as growth in vitro, and transcriptional activity, expressed as the relative rate of transcript elongation. 12

2 Volcano plot of RNAseq data showing the differential expression of compensated genes. 13

3 Volcano plot of SWATH-MS data showing the differential expression of compensated genes. 14

4 Clustering of expression data for the evolutionary strain set. 15

5 Histogram of log-fold changes for genes of significantly enriched genesets in the signature of compensation. 16

6 Genes affected by compensation show congruous expression differences in a simple *rpoB*versus wild type comparison for both the RNA and protein compartments. 17

7 Maximum-likelihood tree of a sampling of *Mtb* genetic diversity. 18

8 The signature of compensation is not shared across strains. 19

9 Genetic distance does not correlate with expression distance imposed by the introduction of the Ser450Leu mutation in RpoB. 20

10 Dissimilarity of protein but not transcript abundances in different backgrounds correlates well with the relative fitness of RpoB^{Ser450Leu} mutants. 21

11 RpoB^{Ser450Leu} imparts an increased investment in proteins relevant to carbohydrate and amino acid metabolism across strains. 22

12 Net proteome cost based on label free quantification (LFQ) estimates for each protein and their constituent amino acid composition is higher overall in RpoB^{Ser450Leu} mutants compared to their drug susceptible ancestors. 23

13 Ribosomes represent approximately 4.2% of the proteome and their level does not correlate with relative fitness of *rpoB* mutants. 24

14 RNA polymerase represents approximately 1.0% of the proteome and their level does not correlate with relative fitness of *rpoB* mutants. 25

15 The chaperone protein DnaJ2 represents a small proportion of the proteome and its level does not correlate with relative fitness of *rpoB* mutants. 26

List of Tables

1 **Growth parameters of clinical isolates of *Mtb*** 7

2 **Correspondence between differential expression in the RNA and protein compartments of rifampcin-resistant *Mtb*** . . . 8

Contents

1 Identifying the basis of RpoB^{Ser450Leu} fitness cost. 5

1.1 General overeview of data 5

1.2 Definining the signature of compensation 5

1.3 Estimating the impact of compensation 6

1.4 Iron homeostasis appears to be a key process affected by compensation 6

2 Epistasis of RpoB^{Ser450Leu} 8

2.1 Genetic distance is independent from expression distance 8

Note on the supplementary material

We added two short sections before the supplementary figures. These sections are meant to complement the text in the main body of the paper. We have decided to include them here to maintain a more focused flow to the paper, while not omitting observations that we thought were pertinent and potentially interesting.

1 Identifying the basis of RpoB^{Ser450Leu} fitness cost.

As we point out in the main text, we expect that the physiological changes incurred by the fitness cost of RpoB^{Ser450Leu} are likely to manifest as deviations in gene expression. And because we know that the secondary mutation in RpoC: Leu516Pro, does have a compensatory role, we expect that comparative analysis will allow us to define the subset of expression changes that are most relevant to the understanding of fitness cost of rifampicin resistance. We used the global profiling tools of RNAseq and SWATH-MS to achieve this.

1.1 General overeview of data

Overall, we found a very high degree of inter-sample similarity – mean Spearman correlation coefficient (ρ_s): 0.98 ($\rho_s$min: 0.94 – $\rho_s$max : 1.00) for RNAseq and 0.97 (0.93 – 0.99) for SWATH-MS. Hierarchical clustering revealed little sample structure in RNAseq data, while SWATH-MS data could be separated into two clear clusters based on the presence or absence of the rifampicin-resistance mutation: see Supplementary Figure 4. Combined, these observations suggest that the *rpoB*Ser450Leu mutation causes a subtle global perturbation of gene expression, which impacts the protein compartment in a more idiosyncratic way.

1.2 Definining the signature of compensation

Next, we used differential expression analysis to identify the functional consequences of rifampicin-resistance. Based on the data overview, we expected that an RNAP mutation would have a pleiotropic effect, albeit one of small magnitude. We envisaged that only a subset of all expression differences specific to RifR would coherently point to a likely biological basis for the reduced growth rate. We hypothesised that this subset would be characterised by a reversal of RpoB^{Ser450Leu}-mediated dysregulation through the phenotypic effect of RpoC^{Leu516Pro}. We named this trend a "signature of compensation", see Figure 2B in the main text, and we derived it by identifying genes that are uniquely differentially expressed in RifR compared to the other three strains in our dataset. To maximise the probability of identifying the signature of compensation, we chose an inclusive definition of differential expression: an adjusted p-value of less than 0.05 for the negative binomial or linear mixed models for transcriptomic and proteomic data, respectively (see Methods in the main text). In keeping with our inclusive approach, we also deliberately did not use an effect size threshold (e.g. minimum log-fold change). Using these criteria, we identified 536 transcripts that could be involved in the cost of resistance. 289 transcripts were less abundant and 247 were more abundant in RifR compared to the other samples (see Figure 2A in the main text

and Supplementary Figure 2). To assess the probability of detecting this many differentially expressed genes by chance, we scrambled the sample labels and repeated the comparison 1,000 times. 70% of the simulated comparisons did not lead to the identification of any differentially expressed genes and the empirical 95% confidence interval spanned 0-95 significantly differentially expressed genes. 536 genes represented the most extreme outcome among the comparisons. The pattern was similar for proteomic data: we found 536 proteins that showed a significant signature of compensation in RifR (260 more and 276 less abundant proteins, see Supplementary Figure 3). 92.8% of the iterations had no significant changes. Our observed effect size lay outside the 95% confidence interval for randomised simulations of proteomic data (95%CI: 0-1 differentially expressed genes).

1.3 Estimating the impact of compensation

The impact of individual mutations on the overall expression profile of a bacterial strain can be estimated by fitting a linear model to the log of the fold changes of treatments of interest [1]. The slope of the fitted model should equate to 1 if the gene expression is fully restored or approach 0 if there is no correlation or effect. Any deviation from 1 should therefore be proportional to the disruption of gene expression. We used this approach to quantify the extent to which the acquisition of RpoC^{Leu516Pro} mitigates the expression differences caused by the gain of RpoB^{Ser450Leu} for both RNAseq and SWATH-MS data. Overall, comparing expression changes of RifR versus RifRevo and RifR versus DS gave us a slope of 0.43 and 0.30 for RNAseq and SWATH-MS data, respectivelly - leading us to conclude that the introduction of RpoC^{Leu516Pro} corrects 43% and 30% of the aberrant expression in the two compartments. Further refining the model by fitting the model exclusively on the genes involved in the signature of compensation, we can see that the slope for this subset of genes is closer to 1: 0.607 and 0.718 for RNAseq and SWATH-MS, respectivelly.

1.4 Iron homeostasis appears to be a key process affected by compensation

Gene set enrichment analysis of the transcriptomic data pointed to iron homeostasis being disproportionally affected. Specifically, it indicated a higher expression of genes that are repressed by the iron-dependent regulator (IdeR, Rv2711) in iron replete conditions (22 significantly affected genes out of 40 in the gene set, Fisher's exact test, Odds ratio = 4.29, p-value = 8.20×10^{-7}, see Supplementary Figure 5). Among them, there was a significant enrichment of genes involved in polyketide and non-ribosomal peptide synthesis, which include the biosynthetic

Supplementary Table 1: Growth parameters of clinical isolates of *Mtb*. Estimates were derived from a mixed effect linear regression.

	Wild type		RpoB^{Ser450Leu}		
	Gen. time [h]	95% CI	Gen. time [h]	95% CI	Fitness cost [%]
N0052	23.9	22.4-25.7	28.2	25.4-31.7	15.2
N0072	25.6	23.1-28.8	29.5	26.6-33.1	13.2
N0145	26.4	23.3-30.4	27.9	24.5-32.3	5.5
N0155	31.0	27.8-35.1	31.6	28.7-35.3	2.0
N0157	22.7	20.8-25.0	30.5	27.5-34.2	25.6

machinery for the sole *Mtb* siderophore: mycobactin (17/41 genes, Fisher's exact test, Odds ratio = 3.20, p-value = 2.30×10^{-4}). Expression changes in protemic data showed little specific gene set enrichment; however, the key trends corroborated the observations we made with transcript analysis. Specifically, proteins involved in the response to iron ion starvation, which include three proteins belonging to the mycobactin biosynthesis cluster, were enriched (8/14 genes, Fisher's exact, Odds ratio = 3.13, p-value = 0.014, see Supplementary Figure 5). Similarly, we found the IdeR-responsive iron-repressed proteins to also echo the pattern of expression detected for the transcripts (13/35 genes, Fisher's exact, Odds ratio = 2.04, p-value = 0.042). However, this trend was no longer statistically significant once we accounted for multiple testing. Combined, these results supported our hypothesis that mutations in *rpoB* impart changes to the baseline expression profile of *Mtb*. Furthermore, these changes could be reversed in the presence of a compensatory mutation in *rpoC*, especially on the transcriptional level. While we observed that this effect was relatively broad, encompassing between 15-20% of the measurable transcripts and proteins, we noticed that genes involved in iron homeostasis appeared to be overrepresented among differentially expressed genes. The availability of iron is an essential requirement for *Mtb* growth, both in culture and during infection, and iron acquisition systems are therefore key virulence factors [2;3;4]. Hence, an increased requirement for iron could manifest itself as a loss of relative fitness. The fact that RpoB^{Ser450Leu} led to a modification of the expression of genes involved in iron homeostasis and that RpoC^{Leu516Pro} reversed the effect, provides a compelling alternative mechanism underpinning the apparent fitness cost of rifampicin resistance.

Supplementary Table 2: Correspondence between differential expression in the RNA and protein compartments of rifampicin-resistant *Mtb* RpoB^{Ser450Leu} mutants were compared to their cognate wild type ancestor to identify differentially expressed genes.

	Differentially expressed transcripts	Differentially expressed proteins	Overlap
N0052	41	983	14
N0072	464	1094	138
N0145	3	2	0
N0155	421	654	74
N0157	236	1784	100

2 Epistasis of RpoB^{Ser450Leu}

Rose *et al.* [11], previously showed that the genetic distance between two *Mtb* strains correlates with their baseline gene expression. This phenomenon might be underlying the differences we observed in our system as well. Starting from this possibility, we expected that correlating *rpoB* mutant-imposed expression differences with the corresponding genetic distances could be used to shed light on the forces that impact gene expression in drug-resistant strains.

2.1 Genetic distance is independent from expression distance

We hypothesised that a positive correlation between genetic distance and expression distance would provide compelling evidence that the response to *rpoB* mutations is modulated by the constellation of mutations that a strain accumulated over its evolutionary history. If true, this result would support the notion that strains belonging to specific lineages or sub-lineages respond to resistance mutations in a common way: a consequence of this being that some backgrounds are more likely to develop resistance, as has been suggested for the Lineage 2 - Beijing family of strains [13;14]. The absence of correlation on the other hand could mean either that the impact of the *rpoB* mutation is independent of genetic distance, or that the modulation of resistance-driven changes in gene expression is done by mutations that were acquired more recently in evolutionary time, making the perturbation of each strain unique. We can discern between the two by looking at the variability in expression distance. In the former case, we would expect the variance to be low, while in the latter, we would expect a large amount of heterogeneity. In order to test our hypothesis, we needed first define a metric of expression distance. We

complemented our earlier approach based on the overlap of differentially expressed genes by calculating the Hamming distance across genes between strain pairs. We also used a fundamentally different measure of similarity that is rooted in expression quantification, either transcript abundance as calculated with the Transcript Per Kilobase Million (TPM) or protein abundance as defined by the Label Free Quantitation (LFQ; see Methods in the main text for further details). We estimated the relationship between genetic distance and expression distance from the fitted parameters of a linear model and quantified the coefficient of variation (CV) for each comparison (See Supplementary Figure 9. We found a low correlation between genetic distance and expression distance across all metrics (R^2: 0.05 – 0.13). The CV for both transcripts and proteins was large on the level of significant gene overlap: 120.3% and 87.2%, respectively, but lower on the level of global metrics of distance. This outcome lead us to conclude the following things:

1. **On the level of significantly affected single genes, each strain is affected in its own private way.** This is corroborated by the fact that there are no mRNAs that are significantly affected by RpoB^{Ser450Leu} in all tested backgrounds (see main text Figure 5A). Similarly, there are large discrepancies among proteomic profiles as well (see main text Figure 5B). Some strain have more than 50% of their proteome affected (N0157) while others see a significant effect for only 2 proteins (N0145).

2. **At a global level, the disimilarity between any two RpoB^{Ser450Leu} effects is stable across genetic distances.** The average divergence of two RpoB^{Ser450Leu} mutants includes about 7.8% of the transcriptome and 24.8% of the proteome.

3. **The proteome sees greater disruption than the transcriptome.** In addition to the fact that the divergence of *rpoB* mutations in two different backgrounds affects a larger proportion of proteins, the number of significantly affected proteins in at least one strain (1914) is much higher than the equivalent measure for mRNAs (958).

References

[1] A. Rodriguez-Verdugo, *et al.* (2016) First-Step Mutations during Adaptation Restore the Expression of Hundreds of Genes. Mol Biol Evol 33:25–39.

[2] C.M. Jones, *et al.* (2014) Self-poisoning of *Mycobacterium tuberculosis*by interupting siderophore recycling. PNAS 111:1945–1950.

[3] R.M. Wells, *et al.* (2013) Discovery of a Siderophore Export System Essential for Virulence of *Mycobacterium tuberculosis* PLoS Pathog 9:e1003120.

[4] P.V. Reddy, *et al.* (2013) Disruption of Mycobactin Biosynthesis Leads to Attenuation of *Mycobacterium tuberculosis*for Growth and Virulence J Infect Dis 208:1255–1265.

[5] M.G. Reynolds (2000) Compensatory Evolution in Rifampin-Resistant *Escherichia coli* Genetics 156:1471–1481.

[6] Q. Qin, G.M. Preston and R.C.MacLean (2014) Linking system-wide impacts of RNA polymerase mutations to the fitness cost of rifampin resistance in *Pseudomonas aeruginosa* mBio 5:e01562.

[7] S. Gagneux, *et al.* (2006) The Competitive Cost of Antibiotic Resistance in *Mycobacterium tuberculosis* Science 312:1944-1946.

[8] M.A. Stefan, F.S. Ugur and G.A. Garcia (2018) Source of the Fitness Defect in Rifamycin-Resistant *Mycobacterium tuberculosis*RNA Polymerase and the Mechanism of Compensation by Mutations in the βŚubunit Antimicrob Agents Chemother 62:e00164-18.

[9] S. Borrell, *et al.* (2018) Reference Set of *M. tuberculosis*Clinical Strains: A tool for research and product development. Preprint at biorXiv doi:10.1101/399709.

[10] I. Comas *et al.* (2013) Out-of-Africa migration and Neolithic co-expansion of *Mycobacterium tuberculosis*with modern humans Nat Genet 45:1176–1182.

[11] G. Rose, *et al.* (2013) Mapping of Genotype-Phenotype Diversity among Clinical Isolates of *Mycobacterium tuberculosis*by Sequence-Based Transcriptional Profiling Genome Biol Evol 5:1849–1862.

[12] H. Akashi and T. Gojobori (2002) Metabolic efficiency and amino acid composition in the proteomes of *Escherichia coli*and *Bacillus subtilis.* PNAS 99:3695–3700.

[13] S. Borrell and S. Gagneux (2009) Infectiousness, reproductive fitness and evolution of drug-resistant *Mycobacterium tuberculosis* Int J Tuberc Lung Dis 13:1456–1466.

[14] C.B. Ford, *et al.* (2013) *Mycobacterium tuberculosis*mutation rate estimates from different lineages predict substantial differences in the emergence of drug-resistant tuberculosis Nat Genet 45:784–790.

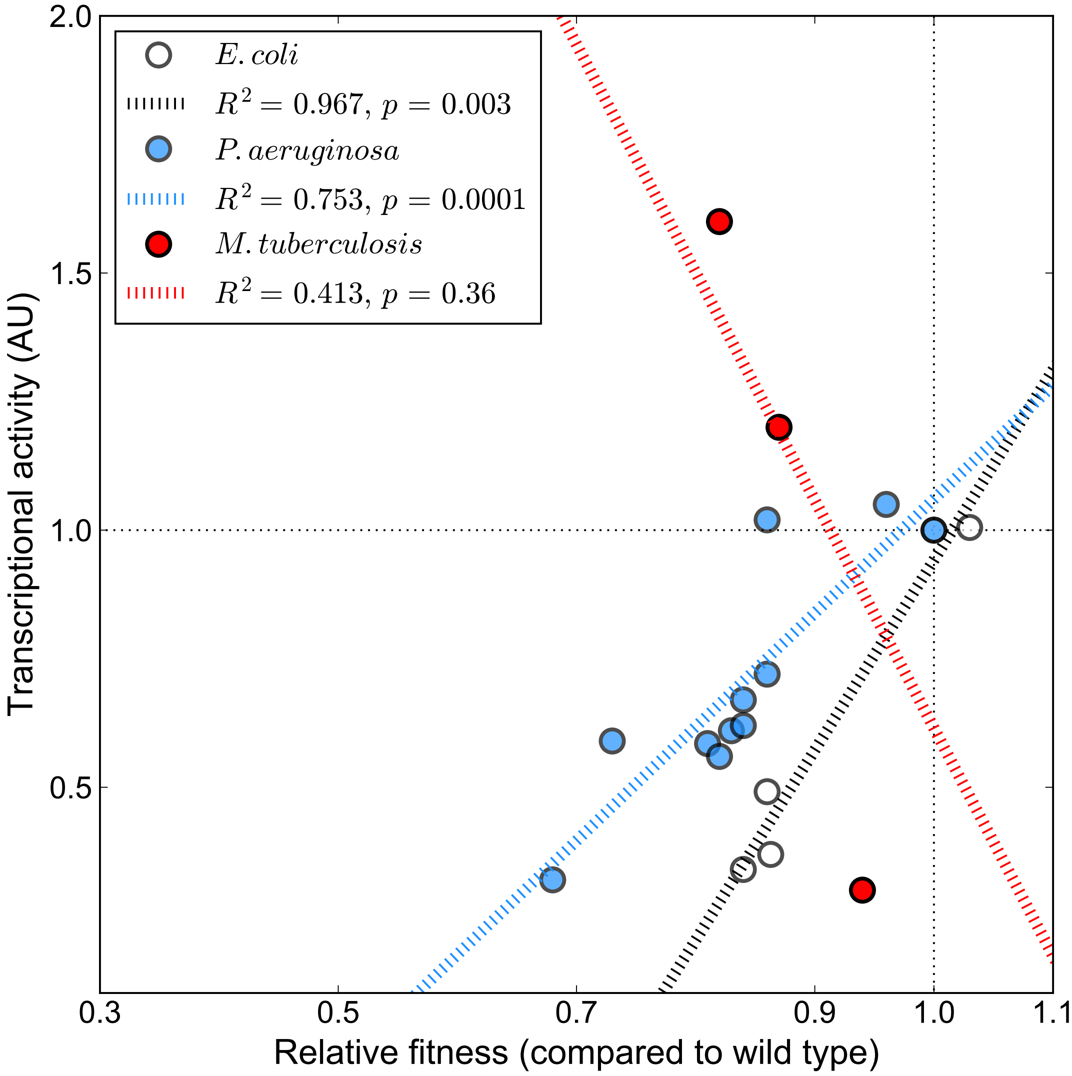

Supplementary Figure 1: Relationship between bacterial fitness, measured as growth in vitro, and transcriptional activity, expressed as the relative rate of transcript elongation. In both cases, the wild type strain is used as reference and the parameters for different RNAP mutants expressed as a proportion thereof. *E. coli* data were obtained from [5], *P. aeruginosa* from [6]. *M. tuberculosis* fitness data were obtained from [7], and the relative transcription rate from [8]. In the case of *E. coli* and *P. aeruginosa* transcriptional efficiency is measured indirectly through the appearance of *lacA* transcript or luciferase-mediated fluoresence, respectivelly. For *M. tuberculosis*, transcriptional efficiency refers to the rate of transcript elongation. R2 and p were obtained by fitting an ordinary least squares linear regression model to the data.

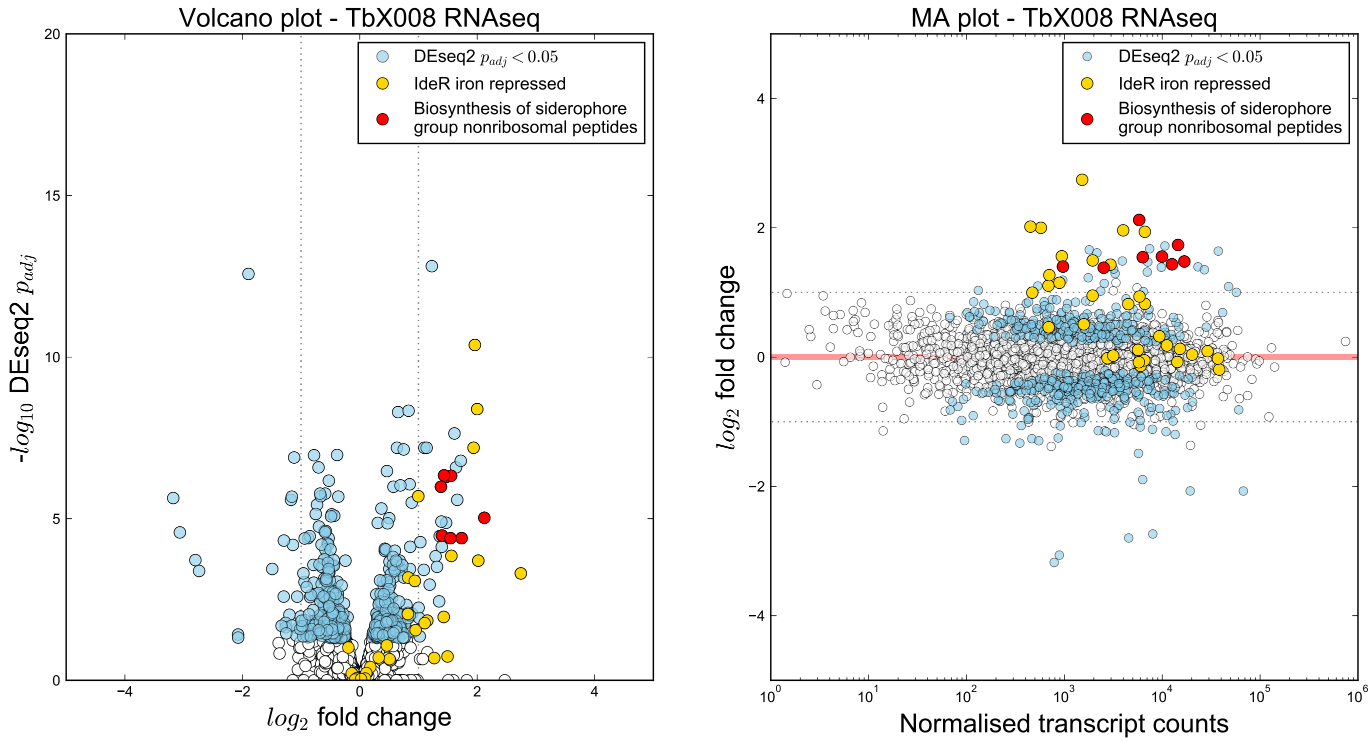

Supplementary Figure 2: Volcano plot of RNAseq data showing the differential expression of compensated genes. We consider all genes whose adjusted p-value (p_{adj}) is lower than 0.05 to be differentially expressed, independently of effect size. Members of the IdeR regulon are highlighted in yellow. Genes involved in the biosynthesis of mycobactin are highlighted in red.

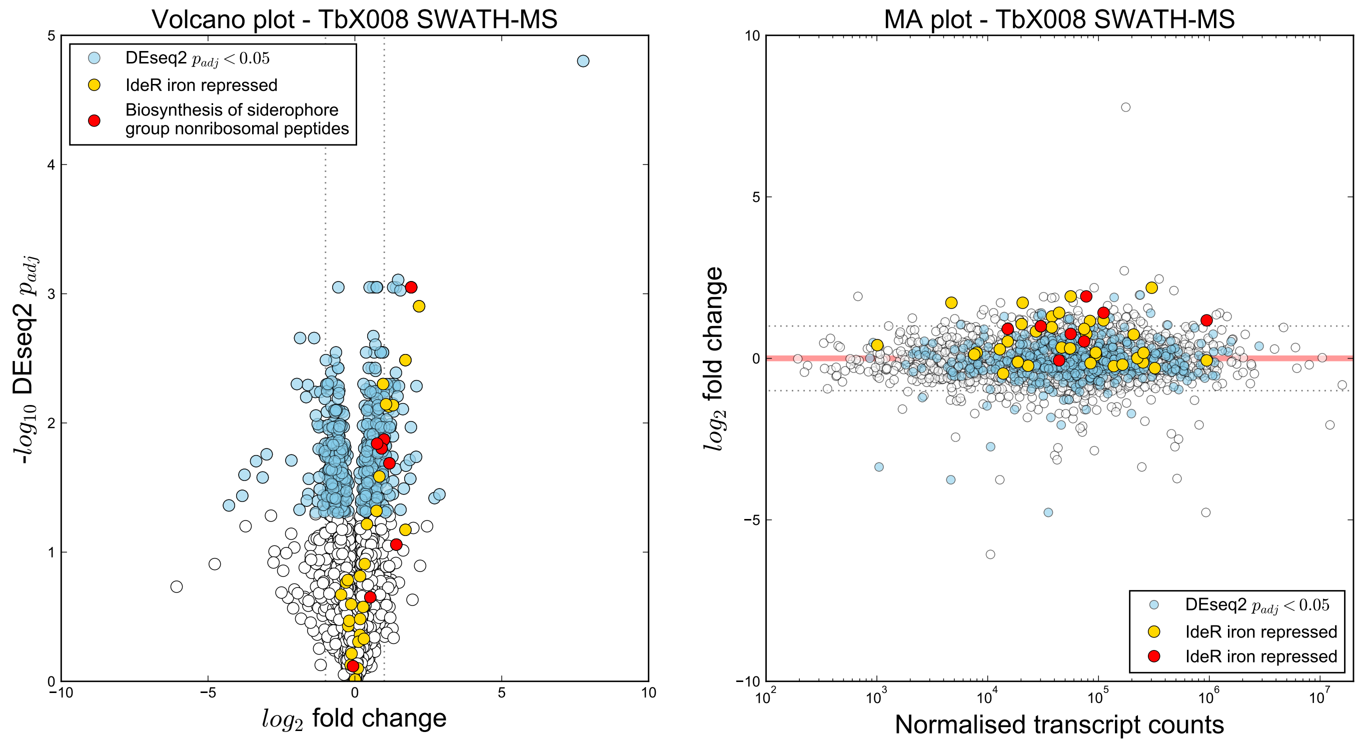

Supplementary Figure 3: Volcano plot of SWATH-MS data showing the differential expression of compensated genes. We consider all genes whose adjusted p-value (p_{adj}) is lower than 0.05 to be differentially expressed, independently of effect size. Members of the IdeR regulon are highlighted in yellow. Genes involved in the biosynthesis of mycobactin are highlighted in red.

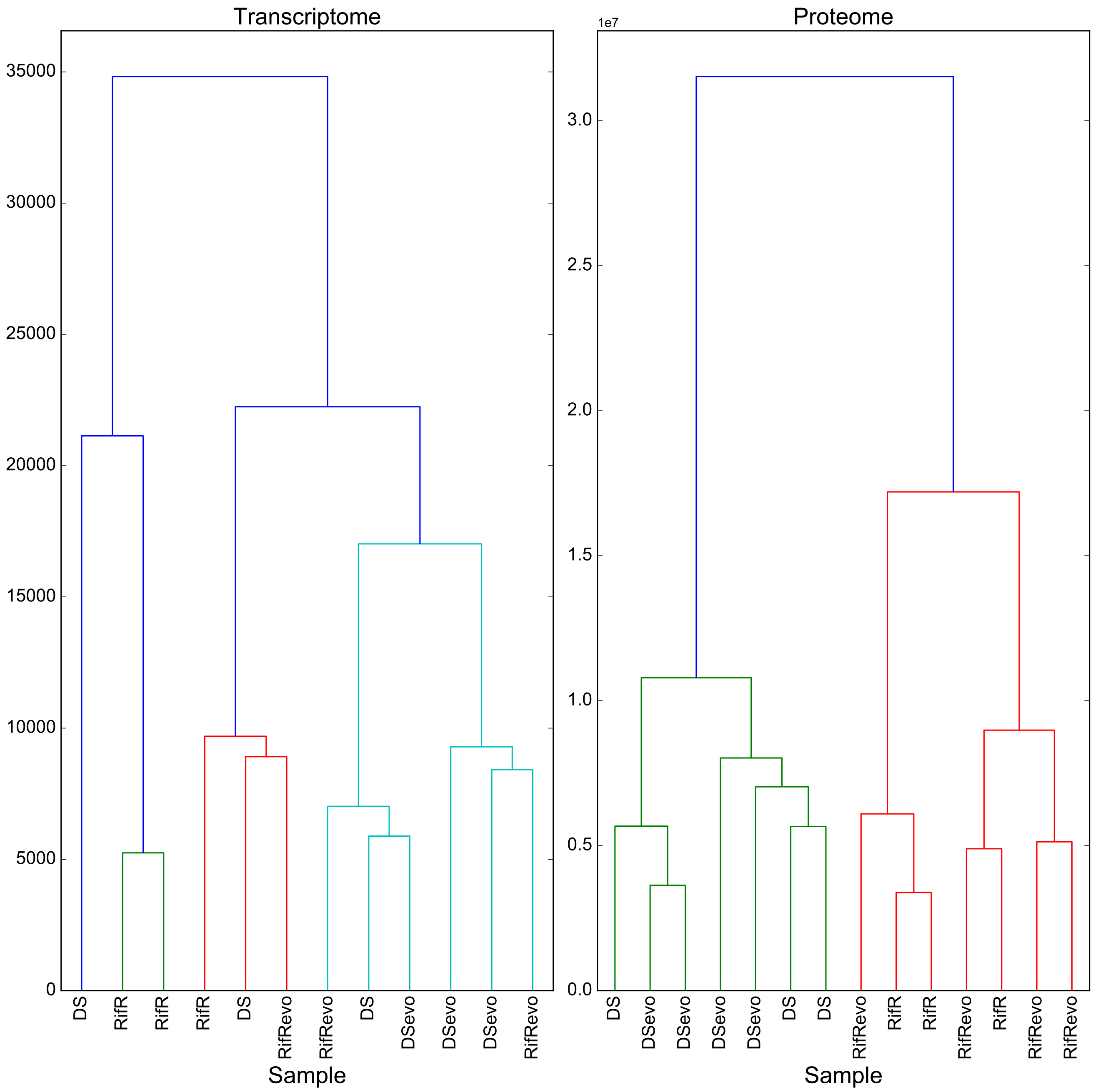

Supplementary Figure 4: Clustering of expression data for the evolutionary strain set. Clustering based on Euclideant distance of TPM for RNAseq (left) and LFQ for SWATH/MS (right), using Ward's Linkage.

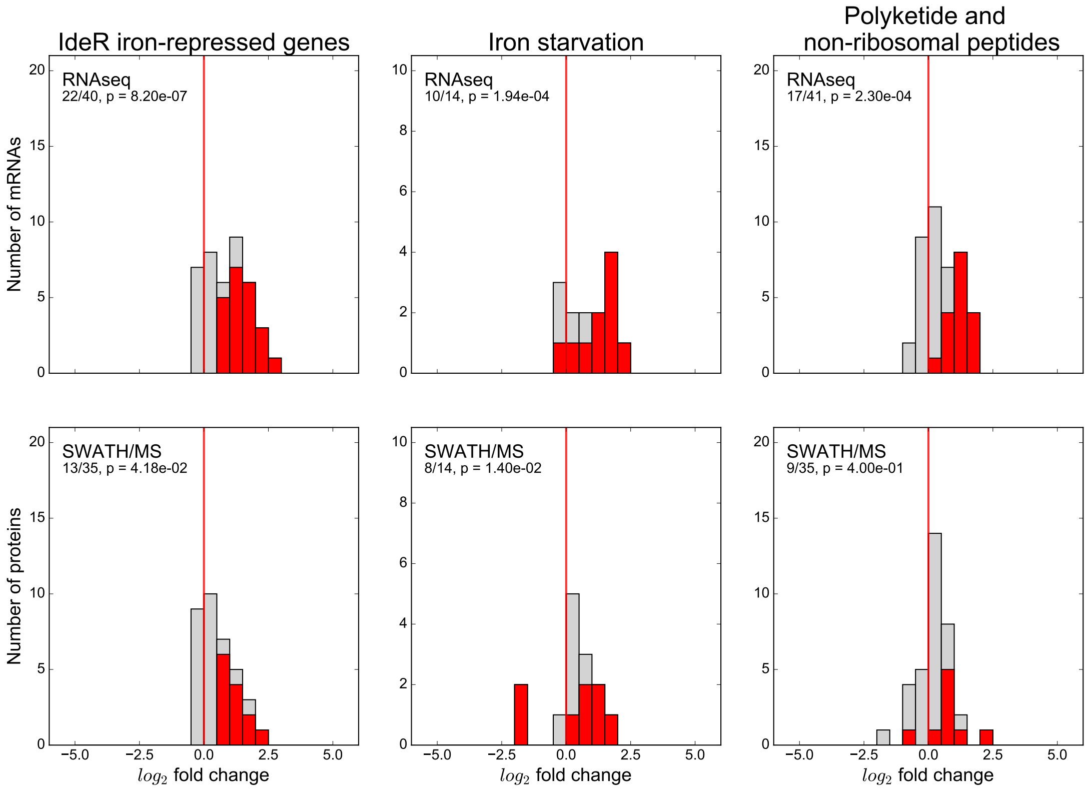

Supplementary Figure 5: Histogram of log-fold changes for genes of significantly enriched genesets in the signature of compensation. Genes that are repressed in an IdeR-dependent way in iron replete conditions are significantly more highly expressed in RifR when compared to their drug susceptible ancestors, as well as their compensated derivatives. Red shading indicates the log-fold change distributions of significantly differentially expressed genes, while grey shading corresponds to non-significant genes. The number of significantly affected genes in a gene set and the size of the gene set is indicated in the top left corner of each panel. The p-value was derived from a Fisher's exact test of enrichment.

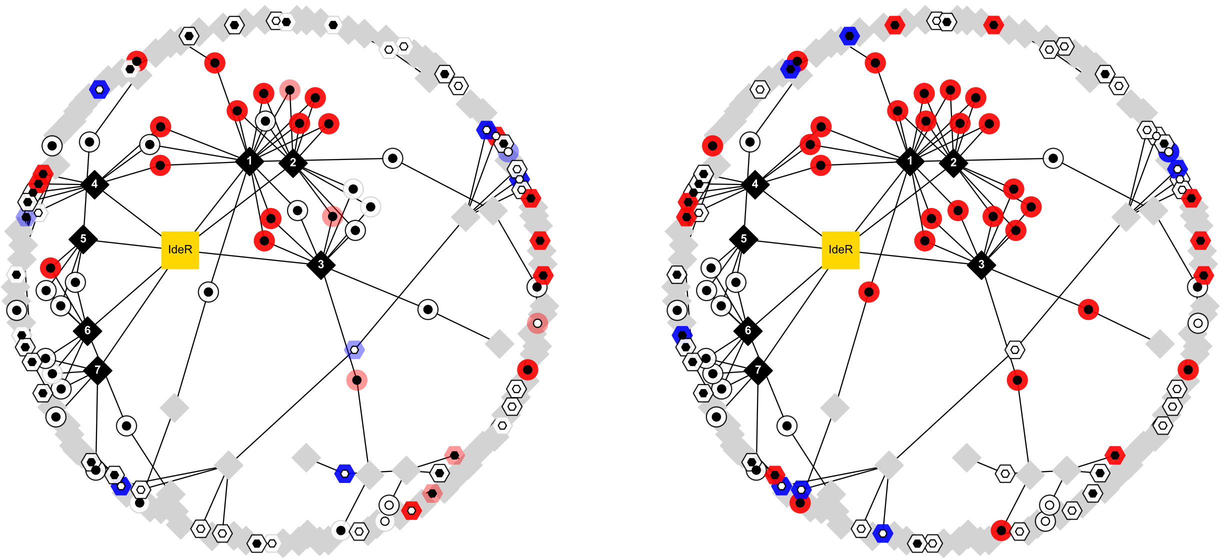

Supplementary Figure 6: Genes affected by compensation show congruous expression differences in a simple *rpoB* versus wild type comparison for both the RNA and protein compartments. Most prominent members of the signature of compensation (see Figure 2 in the main text) are similarly differentially expressed on the level of proteins (left panel). In fact, a limited scope of comparison based on only DS and RifR yields results that recapitulate the signature of compensation (right panel).

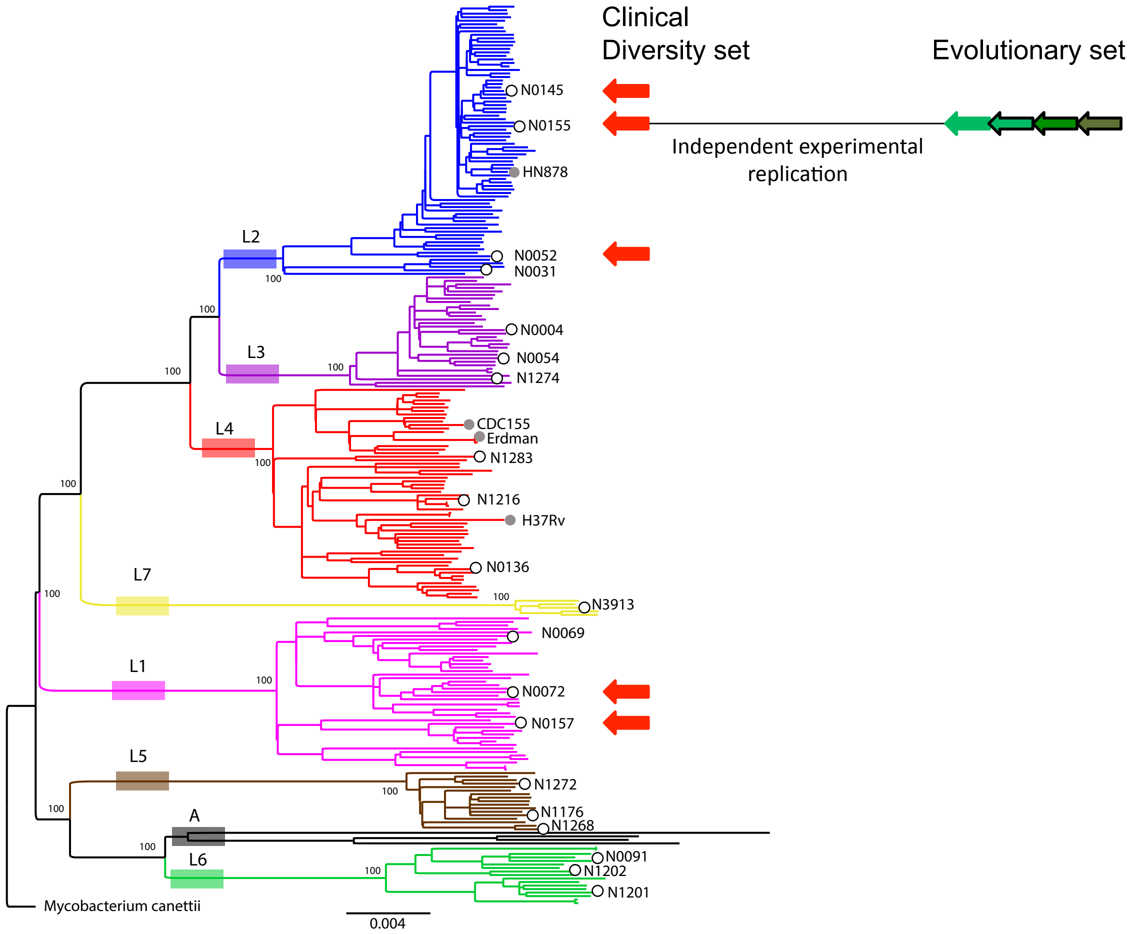

Supplementary Figure 7: Maximum-likelihood tree of a sampling of *Mtb* genetic diversity. Strains used in this study are highlighted with red arrows. Tree adapted from Borrell *et al.* [9]. L1-L7 Phylogenetic lineages 1 -7. 'A' Animal adapted members of the *Mycobacterium tuberculosis* complex. Laboratory strains are indicated with grey circles. Members of the recently proposed Reference Set of Clinical isolates are represented by white circles. The numbers at the tree branching points represent the bootstrap values.

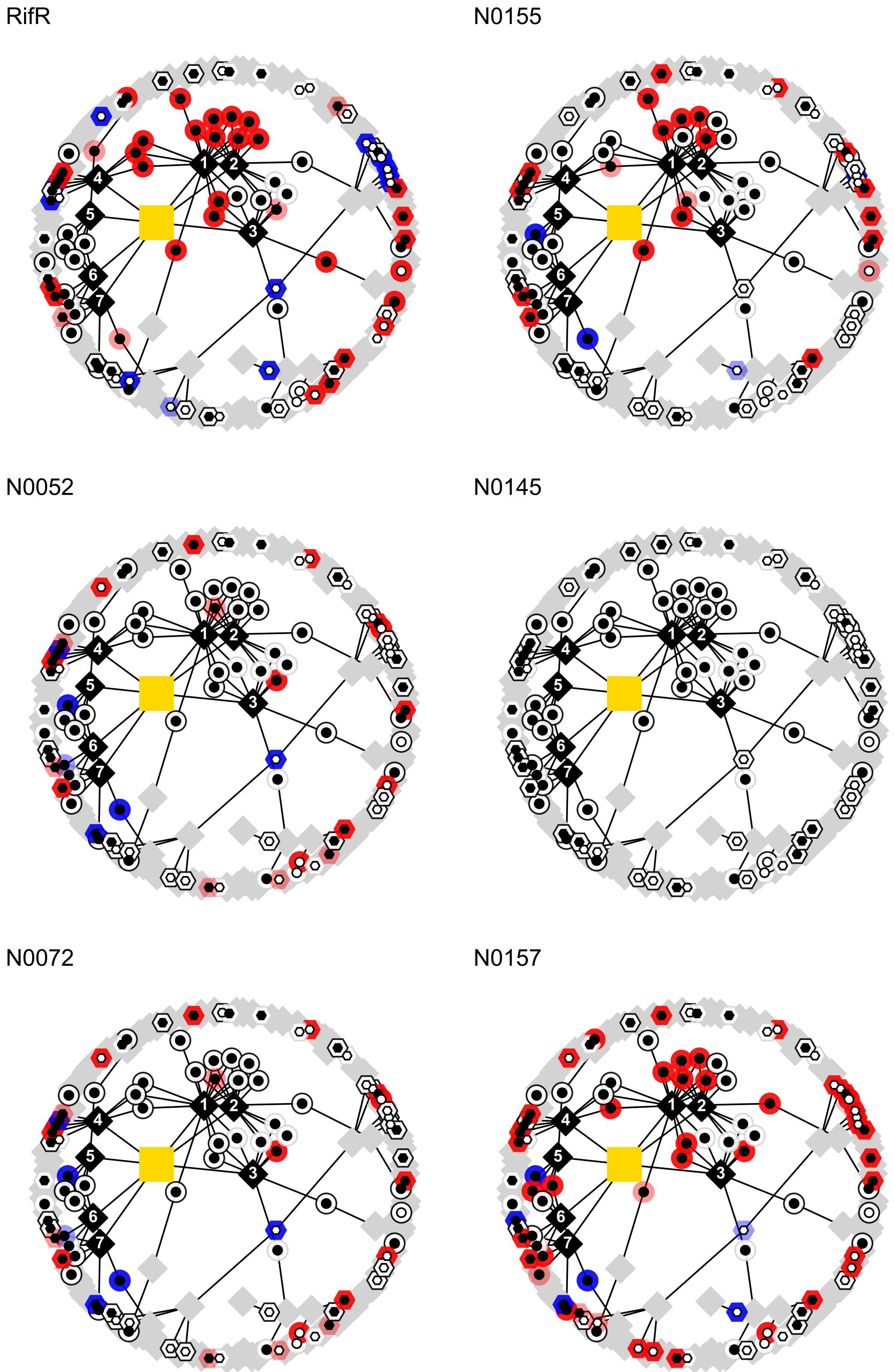

Supplementary Figure 8: The signature of compensation is not shared across strains. Proteomic data echo the observation that IdeR-regulon genes do not play a consistent role in the response to RpoB^{Ser450Leu} (see Figure 4A in the main text).

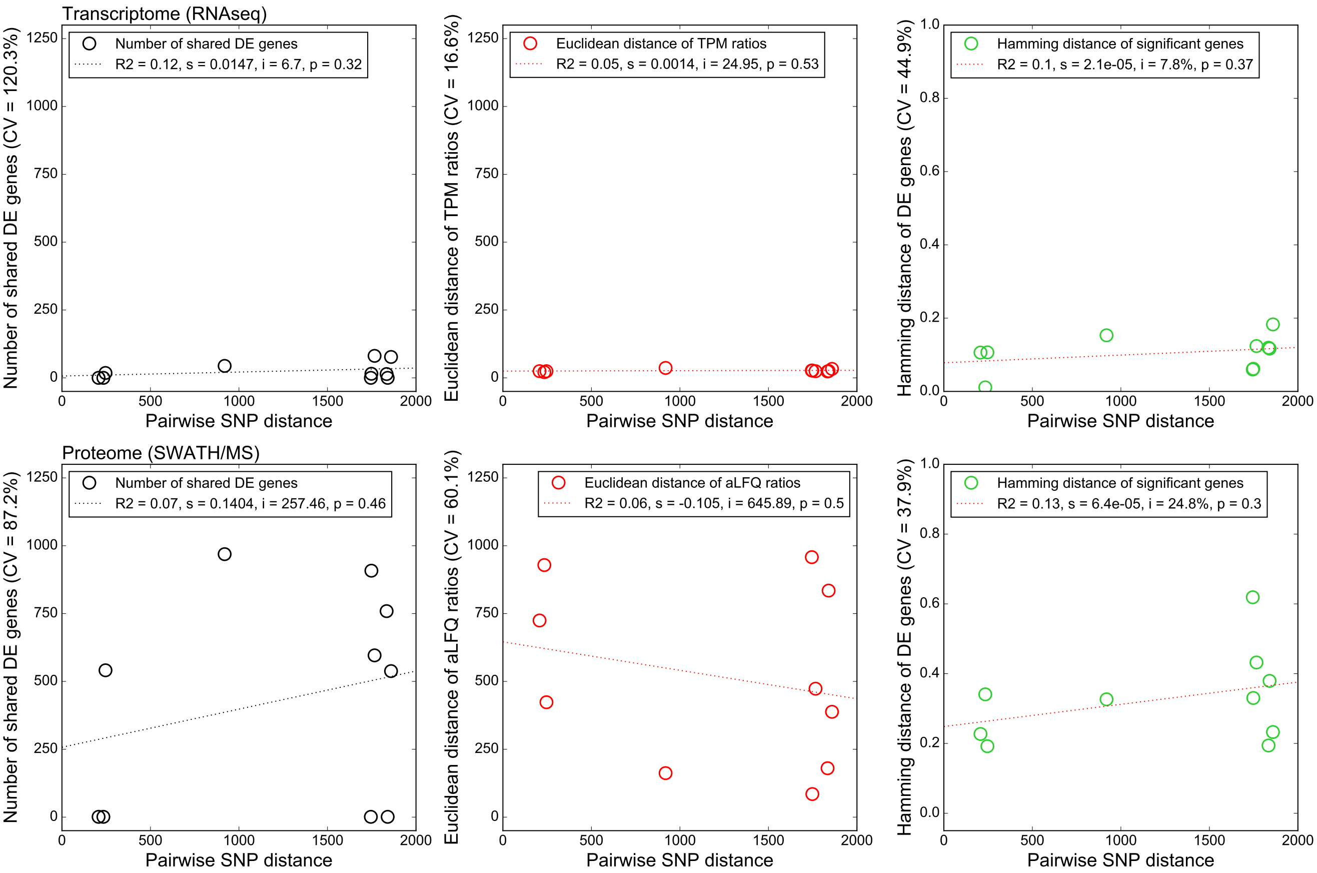

Supplementary Figure 9: Genetic distance does not correlate with expression distance imposed by the introduction of the Ser450Leu mutation in RpoB. There is little overlap in the identity of differentially expressed (DE) genes across different backgrounds. The Euclidean distance of Transcript counts per million bases (TPM) show a more or less constant effect size imposed by the mutation. Hamming and Manhattan distance both show that strains differ based on approximately 10% of the total complement of expressed genes. CV is the coefficient of variance: $\mu\sigma \times 100\%$, the parameters of the linear regression model are: R2 is the square of the Pearson coefficient, s is the slope and i the intercept, p refers to the fit of the linear regression model.

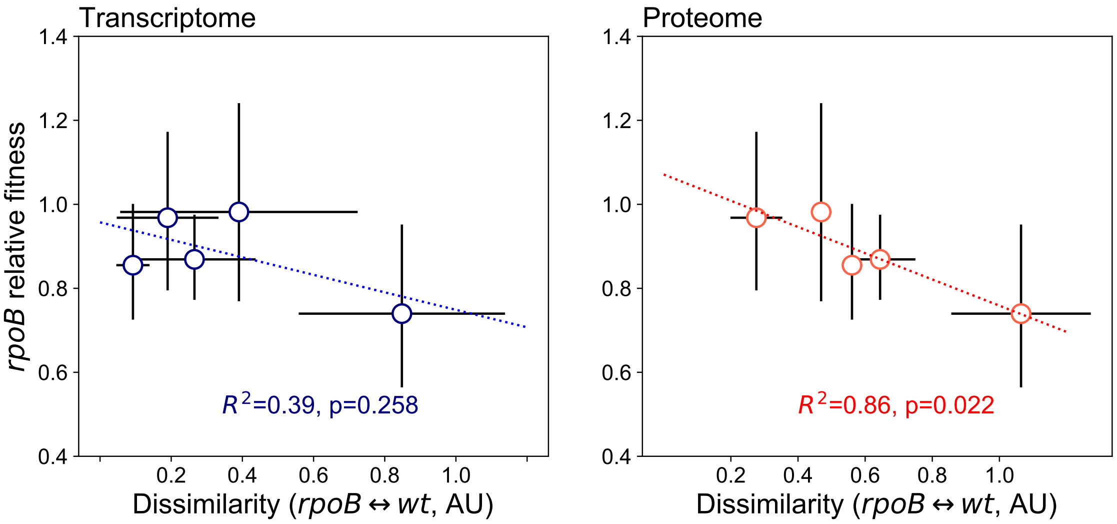

Supplementary Figure 10: Dissimilarity of protein but not transcript abundances in different backgrounds correlates well with the relative fitness of RpoB^{Ser450Leu} mutants. The dissimilarity represents the mean Euclidean distance between TPM and LFQ data from a rifampicin-resistant mutant and its cognate wild type ancestor. The dotted line shows the fit of an ordinary least squares linear regression from which we obtained the R^2 and p-value. The Spearman's correlation (ρ_s) for the data is $\rho_s = -0.10$, p $= 0.87$ for the transcriptome and $\rho_s = -0.80$, p $= 0.10$ for proteomes.

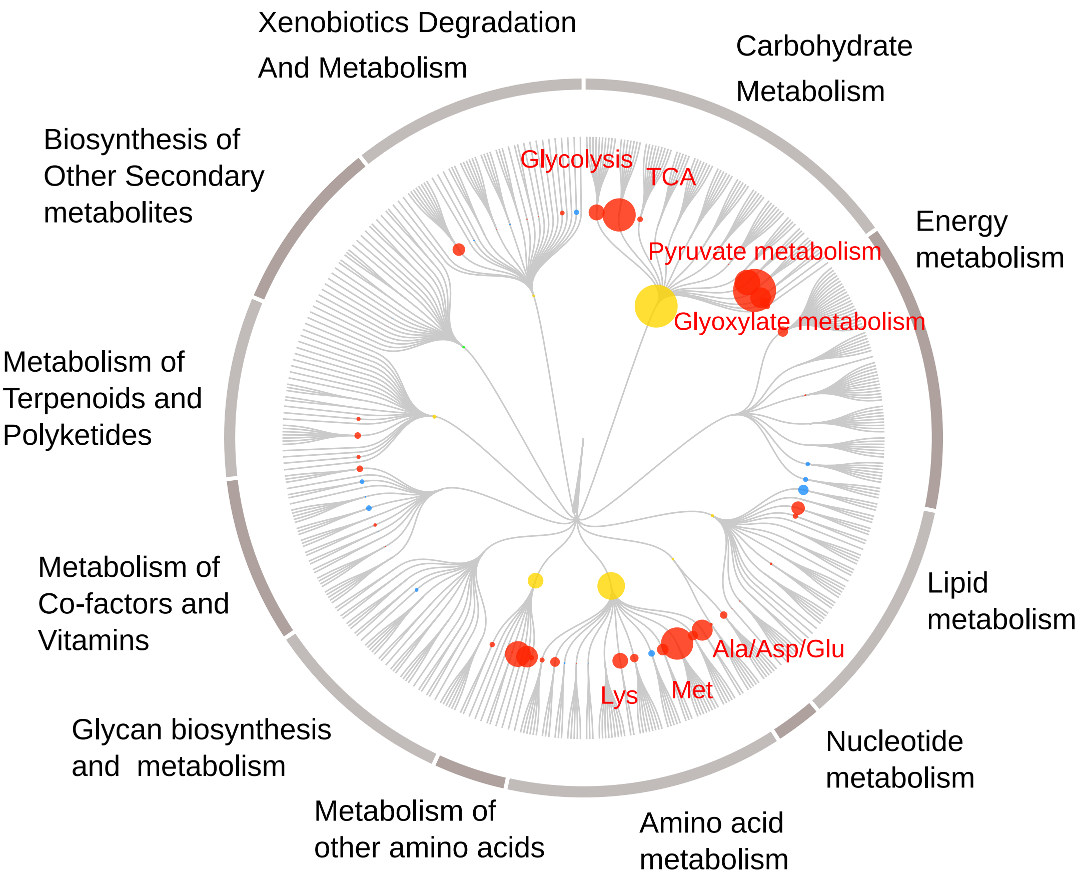

Supplementary Figure 11: RpoB^{Ser450Leu} imparts an increased investment in proteins relevant to carbohydrate and amino acid metabolism across strains. Based on Figure 5B in the main text, as many as 480 proteins are significantly differentially expressed in four of the five tested RpoB^{Ser450Leu} mutants compared to their cognate wild type strains. Most of these proteins were upregulated. We used the SWATH-MS LFQ data, to estimate the magnitude of this expression and applied the FuncTree visualization tool to identify which physiological process is being affected. The physiological processes are represented by each leaf of the dendrogram and are derived from the Kyoto Encyclopedia of Genes and Genomes (KEGG). The radius of the circle provides the relative magnitude of the effect, the absence of a circle indicates no difference. The color of the circle on the outer leaves indicates either a lower (blue) or higher (red) allocation to a process based on LFQ.

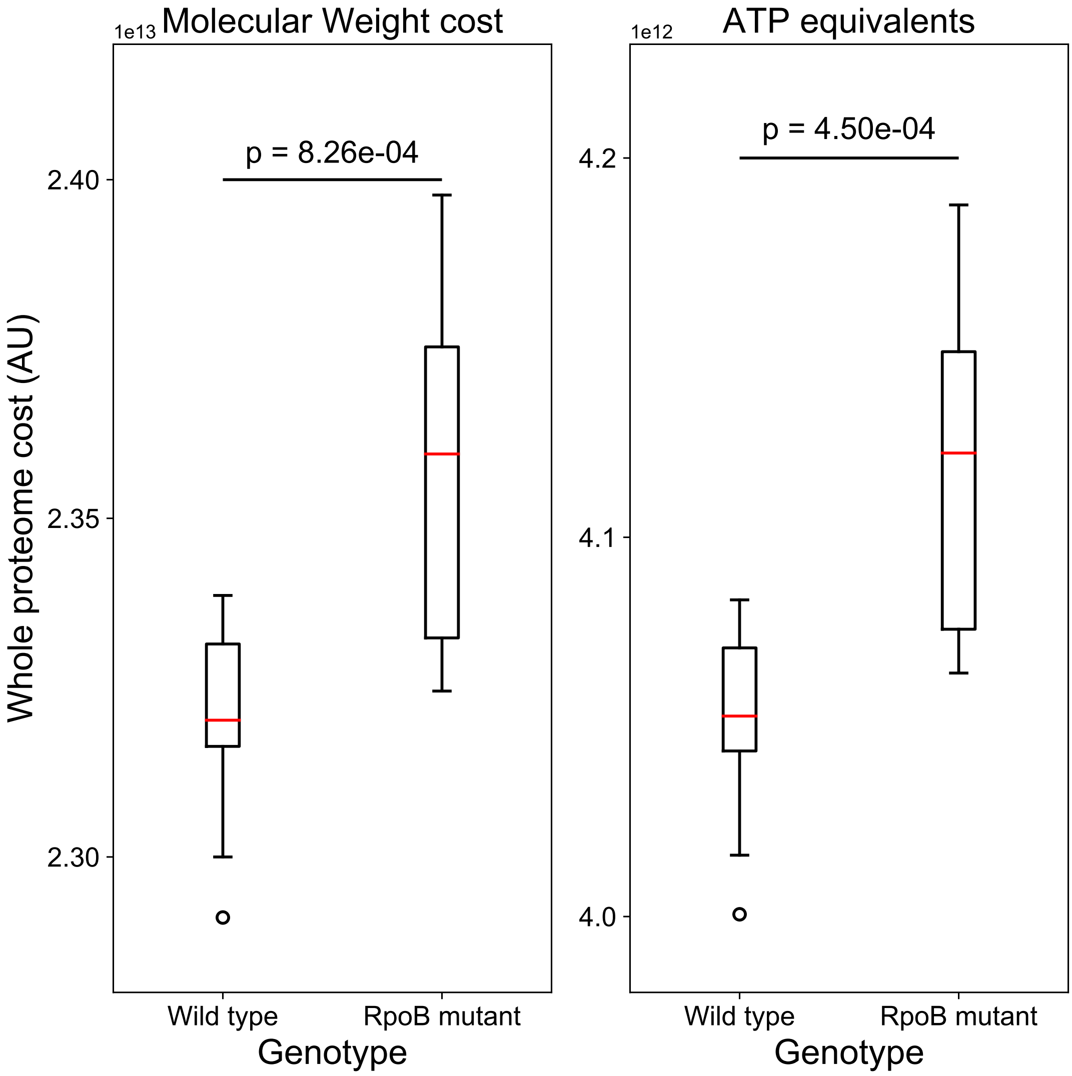

Supplementary Figure 12: Net proteome cost based on label free quantification (LFQ) estimates for each protein and their constituent amino acid composition is higher overall in RpoB^{Ser450Leu} mutants compared to their drug susceptible ancestors. Costs were estimated using either the molecular weights of the constituent amino acids, or the hypothesised chemical energy (ATP) cost as postulated before[12].

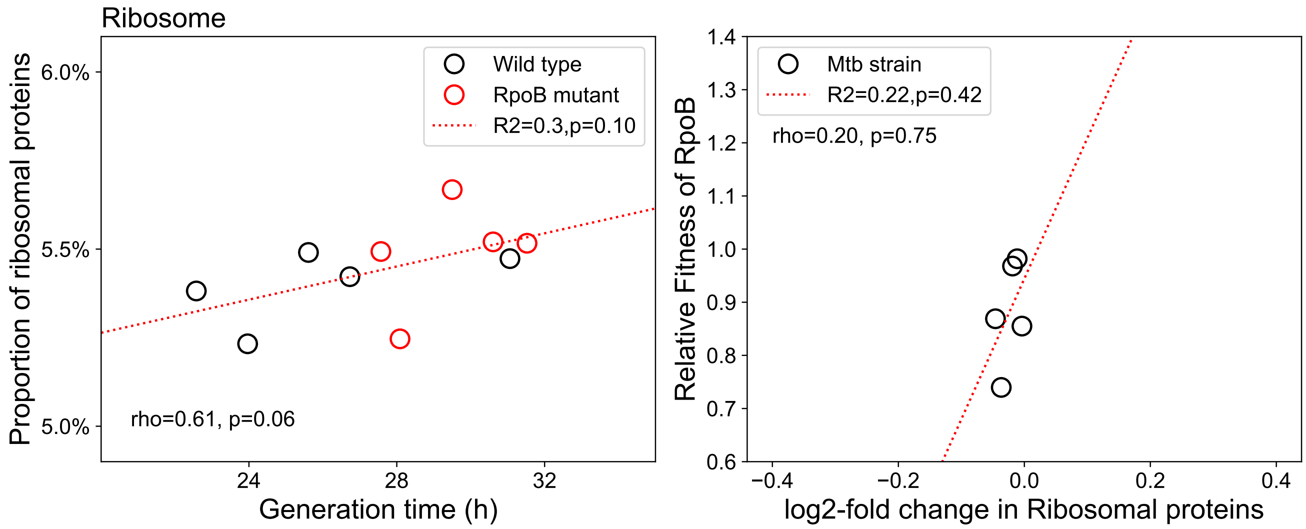

Supplementary Figure 13: Ribosomes represent approximately 4.2% of the proteome and their level does not correlate with relative fitness of *rpoB* mutants. A. Plot of the Proportion of the ribosomal proteins in the proteome estimated from LFQ values derived from SWATH/MS against the strains generation time determined by Mixed effect linear regression of the exponential phase of growth. Dotted line corresponds to the least-squares ordinary linear regression. The R2 and p-value of the regression as well as the Spearman's Rho and its p-value for the regressors are indicated. The proportion of ribosomal proteins was averaged across biological replicates. **B.** Plot of the change in the levels of ribosomal proteins in the proteome estimated from LFQ values derived from SWATH/MS between wild type strains and their RpoB Ser450Leu mutant against the relative fitness of RpoB Ser450Leu mutants determined by Mixed effect linear regression of the exponential phase of growth. Dotted line corresponds to the least-squares ordinary linear regression. The R2 and p-value of the regression as well as the Spearman's Rho and its p-value for the regressors are indicated. The proportion of ribosomal proteins was averaged across biological replicates.

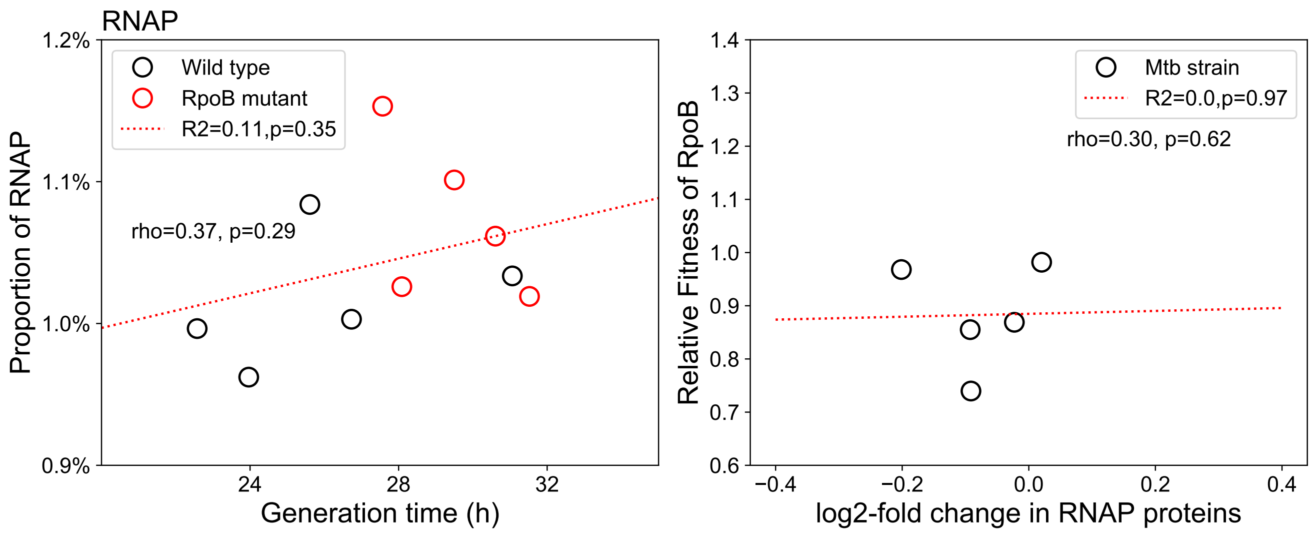

Supplementary Figure 14: RNA polymerase represents approximately 1.0% of the proteome and their level does not correlate with relative fitness of *rpoB* mutants. A. Plot of the Proportion of the RNA polymerase core proteins in the proteome estimated from LFQ values derived from SWATH/MS against the strains generation time determined by Mixed effect linear regression of the exponential phase of growth. Dotted line corresponds to the least-squares ordinary linear regression. The R2 and p-value of the regression as well as the Spearman's Rho and its p-value for the regressors are indicated. The proportion of RNA polymerase core proteins was averaged across biological replicates. **B.** Plot of the change in the levels of RNA polymerase core proteins in the proteome estimated from LFQ values derived from SWATH/MS between wild type strains and their RpoB Ser450Leu mutant against the relative fitness of RpoB Ser450Leu mutants determined by Mixed effect linear regression of the exponential phase of growth. Dotted line corresponds to the least-squares ordinary linear regression. The R2 and p-value of the regression as well as the Spearman's Rho and its p-value for the regressors are indicated. The proportion of RNA polymerase core proteins was averaged across biological replicates.

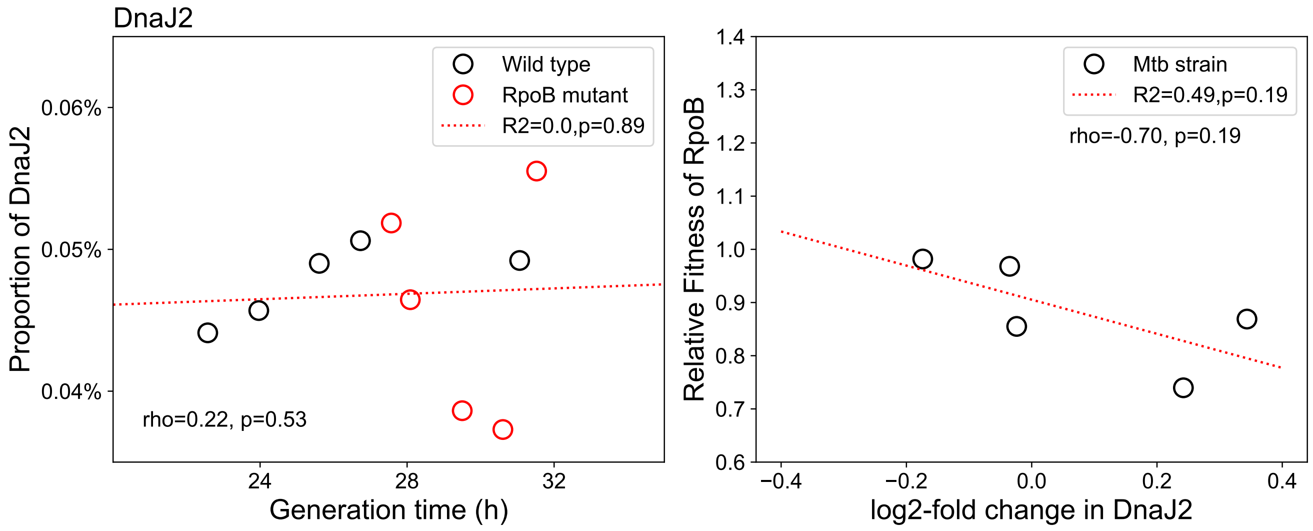

Supplementary Figure 15: The chaperone protein DnaJ2 represents a small proportion of the proteome and its level does not correlate with relative fitness of *rpoB* mutants. A. Plot of the relative abundance of DnaJ2 in the proteome estimated from LFQ values derived from SWATH/MS against the strains generation time determined by Mixed effect linear regression of the exponential phase of growth. Dotted line corresponds to the least-squares ordinary linear regression. The R2 and p-value of the regression as well as the Spearman's Rho and its p-value for the regressors are indicated. The abundance of DnaJ2 was averaged across biological replicates. **B.** Plot of the change in the levels of DnaJ2 in the proteome estimated from LFQ values derived from SWATH/MS between wild type strains and their RpoB Ser450Leu mutant against the relative fitness of RpoB Ser450Leu mutants determined by Mixed effect linear regression of the exponential phase of growth. Dotted line corresponds to the least-squares ordinary linear regression. The R2 and p-value of the regression as well as the Spearman's Rho and its p-value for the regressors are indicated. The proportion of DnaJ2 was averaged across biological replicates.
